# Study on the driving mechanisms of land use change on water yield and carbon storage based on the InVEST-PLUS-GeoDetector model

**DOI:** 10.1038/s41598-026-47409-6

**Published:** 2026-05-20

**Authors:** Jiwei Zhao, Peijin Xiao, Taotao He, Luyao Wang, Yuru Li

**Affiliations:** Water Conservancy College, North China University of Water Resources and Electric Power, Zhengzhou, 450046 China

**Keywords:** Land use transition, Water yield, Carbon storage, InVEST-PLUS-GeoDetector model, Driving mechanisms, Ecology, Ecology, Environmental sciences

## Abstract

Under the dual pressures of global climate change and intensive human activities, the degradation of ecosystem services has become a critical challenge for regional sustainable development. This study aims to investigate the spatiotemporal evolution and driving mechanisms of water yield and carbon storage in the middle reaches of the Yellow River. By integrating the land use transfer matrix, InVEST, PLUS, GeoDetector, and spatial autocorrelation analysis, we assessed the impacts of land use change on water yield and carbon storage from 1985 to 2023. The results indicate that cropland area declined by approximately 15%, while construction land expanded rapidly during the study period, intensifying land use conflicts. Water yield exhibited a pronounced fluctuation–recovery pattern, with a sharp decline of nearly 70% in the early stage followed by a gradual recovery in recent years, reflecting the combined influence of climate variability and human activities. In contrast, carbon storage showed a steady increasing trend associated with vegetation restoration, rising by about 2% over the study period. Driving factor analysis revealed that interactions between vegetation conditions (NDVI) and human activity indicators played a dominant role in shaping ecosystem service dynamics (q > 0.85). A clear trade-off relationship was observed between water yield and carbon storage, indicating the tension between vegetation restoration and regional water availability. Based on these results, this study recommends implementing zonal management strategies, strengthening vegetation restoration in ecologically fragile areas, and optimizing land use structures in urban expansion zones to promote the coordinated enhancement of ecosystem services, thereby ensuring ecological protection and high-quality development in the Yellow River Basin.

## Introduction

Driven by both climate variability at the global scale and the intensification of anthropogenic pressures, the decline in ecosystem services has become a critical obstacle to achieving sustainability worldwide^1^. According to the report of the Intergovernmental Science-Policy Platform on Biodiversity and Ecosystem Services (IPBES), the extent and condition of global natural ecosystems have declined by an average of 47% relative to the baseline, with agricultural expansion being one of the primary drivers of forest fragmentation and biodiversity loss^2^. Meanwhile, the problem of global land degradation continues to worsen, underscoring the urgent need to strengthen ecological restoration and sustainable management^3^. Ecological restoration planning toward land degradation neutrality is considered a key strategy to address this challenge, as it enhances ecosystem stability and promotes sustainable development^4^.

The middle reaches of the Yellow River represent a typical intersection of ecologically fragile zones and areas of rapid socio-economic development in China, where ecosystem services play a strategically vital role in the national ecological security framework^5^. On the one hand, this region serves as an essential ecological barrier of the Yellow River Basin, critical for maintaining water conservation, soil retention, and climate regulation^6^. On the other hand, it is also a core area of agricultural production and rapid urbanization, characterized by high population density and intensive land use^7^. Economic activities exert persistent pressure on natural ecosystems, leading to ecosystem service degradation and uneven spatial distribution^8^. Located in an arid to semi-arid transition zone, The middle reaches of the Yellow River faces fundamental water scarcity as a critical constraint, where water yield services are directly linked to regional water security. Concurrently, the soil carbon pool of the Loess Plateau constitutes a significant component of the global carbon cycle. Large-scale vegetation restoration initiatives, such as the “Grain-for-Green” program, have been widely reported to influence both carbon storage and regional water consumption. As a result, water yield and carbon storage, which exhibit a characteristic trade-off relationship in the region, have become a key focus for understanding interactions between ecological processes and human activities. Existing studies have demonstrated a significant correlation between land use change and ecosystem degradation in the middle reaches of the Yellow River, with agricultural expansion further intensifying ecological pressures^9^.

Driven by the national strategy of ecological civilization, a series of large-scale projects—such as the Grain-for-Green Program and the Xiaolangdi Water Conservancy Project—have contributed to ecological improvements in certain areas, leading to partial restoration of ecosystem services^10^. However, changes in land use structure have also complicated the trade-offs and synergies among ecosystem services, while conflicts between resource exploitation and ecological protection have become increasingly pronounced in some localities^11^. Balancing regional economic development with ecological conservation has thus become a central issue^12^. Current research mainly focuses on three aspects: (1) assessing the impacts of land use change on ecosystem services, (2) exploring the trade-offs and synergies among different ecosystem services, and (3) examining their spatial patterns^10–13^.

Nevertheless, a critical gap remains in our understanding of the scale-dependent dynamics and underlying mechanisms of ecosystem service trade-offs and synergies. Existing studies often lack a multi-scale analytical perspective that can seamlessly link regional patterns with local heterogeneities. Furthermore, the interactive driving effects of natural and socioeconomic factors, which are essential for revealing the formation mechanisms of these relationships, remain inadequately quantified and understood^14–18^.

To address these gaps, this study integrates multi-source remote sensing data and ground-based monitoring records from 1985 to 2023 to investigate land-use dynamics and ecosystem services in the middle reaches of the Yellow River. An integrated analytical framework combining the PLUS model, the InVEST model, and the GeoDetector method is employed to quantify changes in water yield and carbon storage and to explore their potential driving factors across multiple scales. By providing a long-term and spatially explicit assessment, this study aims to support sustainable land-use management and ecological conservation in the Yellow River Basin and other ecologically fragile regions.

## Research methodology

### Study area overview

**Fig. 1:**
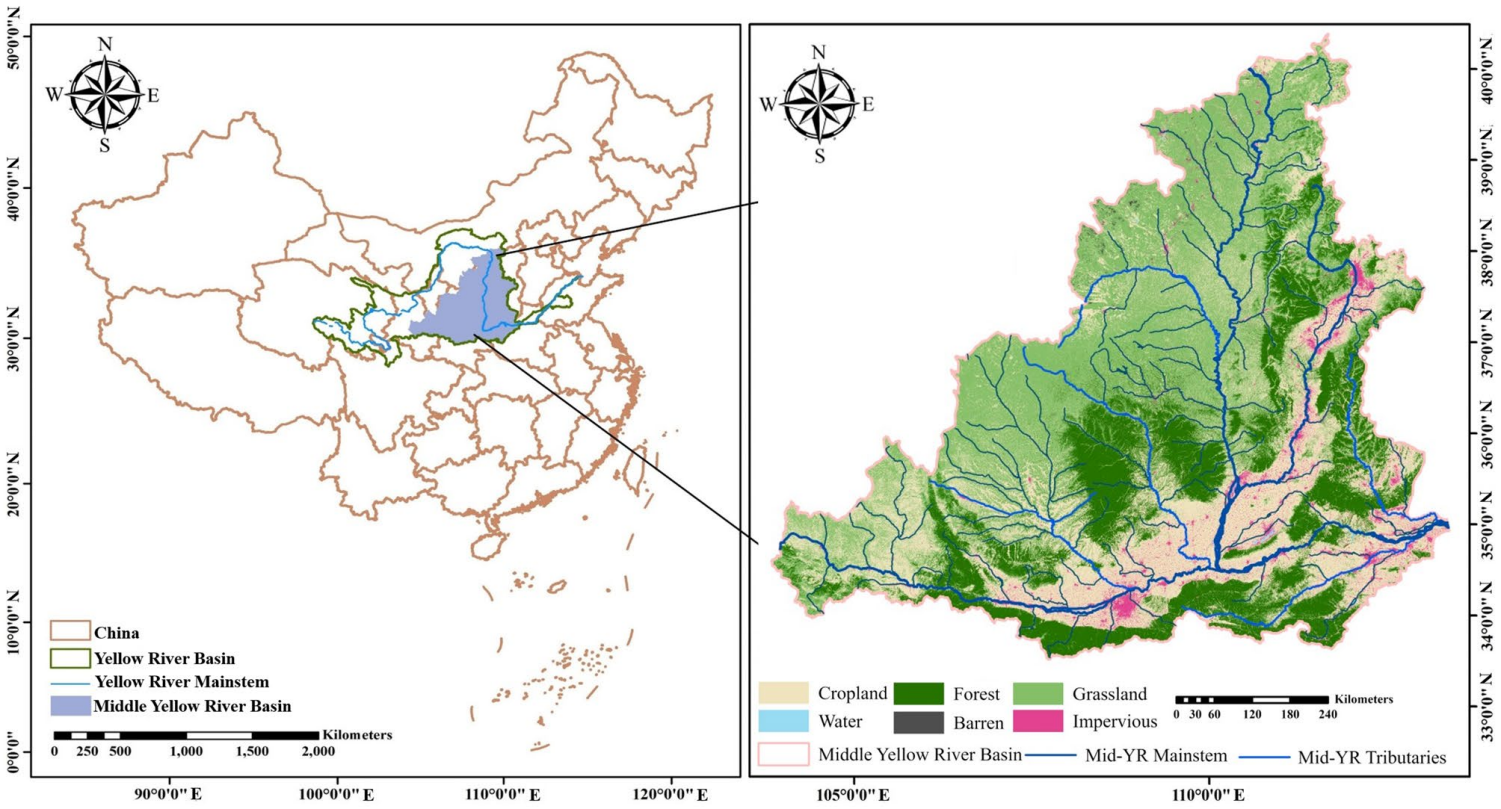
Geographic location and elevation map of the study area This map was created using ArcGIS Pro 3.4 (https://www.esri.com/software/arcgis-pro). The DEM data were derived from the Geospatial Data Cloud (http://www.gscloud.cn), and the administrative boundary data were obtained from the National Geoinformation Public Service Platform (https://www.webmap.cn).

The middle section of the Yellow River lies in northern China, stretching from Hekou in Togtoh County of Inner Mongolia to Taohuayu, situated in Zhengzhou, Henan Province. Geographically, the region lies between 110°–114° E and 34°–39° N (Fig. 1). This study selects the middle reaches of the Yellow River as the research area due to its irreplaceable typicality and uniqueness within the broader river basin and even the national ecological security pattern.

The study area is situated in the core of the Loess Plateau. Acting as a transitional zone between arid and semi-arid regions, its hydrothermal differentiation pattern, combined with the loose loess parent material, collectively leads to severe soil erosion and sensitive water-carbon processes. Concurrently, this area has experienced significant expansion of human activities. The total grain output in Shaanxi Province increased from approximately 7.57 million tons in 1990 to 10.891 million tons in 2000, representing an increase of 43.87%, which reflects the reclamation expansion undertaken to ensure food security. Furthermore, the livestock inventory (in sheep units) in the region rose markedly from 10,192 in 1985 to 111,804 in 2000, a growth of 997% compared to 1985, exerting substantial pressure on the grassland ecosystem. The core conflict between ecological protection, food security, and energy development is prominently manifested here, making it an ideal field for investigating human-environment system interactions.

Compared to the upper reaches—dominated by alpine meadows and water recharge—and the lower reaches—characterized by alluvial plains and flood risks—the central ecological issue in the middle reaches revolves around the synergies and trade-offs within the water-soil-carbon processes. Ecological degradation in the upper reaches primarily manifests as grassland degradation, while the core issues in the lower reaches are water scarcity and pollution. In the middle reaches, however, large-scale vegetation restoration initiatives (e.g., the Grain-for-Green Program), while enhancing carbon sequestration and reducing sediment, may exacerbate regional water scarcity due to their high water consumption. This water-carbon trade-off constitutes the most distinctive and central ecological management dilemma in this region. Therefore, in-depth research on the driving mechanisms of land use change impacts on water yield and carbon storage in the middle reaches of the Yellow River is of paramount importance for the ecological protection and high-quality development of the entire basin.

### Data sources

This study conducted a comprehensive analysis of tributary basins in the middle reaches of the Yellow River based on multi-source datasets, including land-use data, DEM, administrative boundaries, as well as natural environmental and socio-economic data (Table 1).

**Table 1:** Major data sources.

Data Type	Resolution	Source
DEM	90 m	Geospatial Data Cloud (http://www.gscloud.cn/)
Land use data	30 m	Resource and Environmental Science and Data Center (https://www.resdc.cn/)
Precipitation, temperature, potential evapotranspiration	1 km	National Meteorological Science Data Center (http://data.cma.cn)
Soil type, vegetation type, NDVI	1 km	National Tibetan Plateau Science Data Center (http://data.tpdc.ac.cn/zh-hans/)
Nighttime light index, GDP, population	1 km	Resource and Environmental Science and Data Center (https://www.resdc.cn/)
Railways, rivers, administrative boundaries (vector data)	–	National Geoinformation Public Service Platform (https://www.webmap.cn/)

The 1985–2023 time span involves remote sensing data acquired from multiple Landsat sensors (TM, ETM+, and OLI), which differ in spectral response functions, radiometric calibration, and native spatial resolutions. To ensure temporal consistency in land-use classification, the following harmonization steps were implemented: Atmospheric and Radiometric Correction: All Landsat images were subjected to unified atmospheric correction using the LEDAPS (for TM/ETM+) and LaSRC (for OLI) algorithms, which convert digital numbers to surface reflectance and reduce sensor-to-sensor radiometric discrepancies. Spectral Band Harmonization: Due to differences in spectral response between Landsat TM/ETM + and OLI (particularly in the blue, NIR, and SWIR bands), we applied published cross-sensor reflectance conversion coefficients to harmonize OLI reflectance into a TM-consistent spectral space, ensuring comparability across the entire time series. Spatial Resolution Standardization: To achieve spatial consistency, all images were resampled to a uniform spatial resolution of 30 m using the nearest-neighbor method for categorical land-use data. This step prevents spatial artifacts caused by mixed resolutions (e.g., OLI 15 m panchromatic or 30 m multispectral vs. TM 30 m). Temporal Quality Screening: For each target year, multiple candidate scenes were evaluated and cloud-free (< 10%) images were prioritized. When necessary, multi-scene mosaicking and gap-filling procedures were applied to mitigate ETM + SLC-off striping after 2003. These harmonization procedures effectively reduce the temporal inconsistencies caused by different Landsat sensors and ensure that land-use change detection across 1985–2023 is driven by true surface changes rather than sensor variability.

This study selected 1985 to 2023 as the research period, mainly based on the following three considerations:

1. The mid-1980s marks the beginning of consistent, large-scale satellite remote sensing data (e.g., Landsat), which provides reliable and continuous land-use/cover data. This ensures the accuracy and comparability of our starting point and the entire time-series analysis.
2. This period comprehensively spans several critical phases of ecological and socio-economic development in the Middle Yellow River. It includes the initial phase of rapid agricultural expansion and economic development (1980–1990 s), the full implementation of major ecological restoration projects starting around 1999 (e.g., the “Grain-for-Green” Program), and the recent acceleration of urbanization under China’s national strategy for “Ecological Protection and High-Quality Development in the Yellow River Basin.” This allows our study to effectively capture land-use transitions and their ecological impacts driven by different dominant policies.
3. A nearly 40-year time span is sufficient to reveal the long-term trends and lagged responses of ecosystem services (like water yield and carbon storage) to climate change and human activities. It helps us identify characteristic phases such as the “fluctuation–recovery” pattern and provides a more profound understanding of the underlying driving mechanisms.

The temporal segmentation of 1985–2023 in this study is closely aligned with major land-use and ecological policies implemented in China. Specifically, the period before 2000 corresponds to rapid agricultural expansion under food security-oriented policies, during which cropland reclamation was encouraged in the Loess Plateau. Since 1999, the Grain-for-Green Program has been implemented in a phased and regionally differentiated manner, with the middle reaches of the Yellow River serving as a core implementation zone. By 2010, the cumulative converted area in this region accounted for more than 30% of the national total restoration area. After 2012, the concept of “Ecological Civilization” was elevated to a national strategy, further strengthening ecological protection intensity, while the “Yellow River Basin Ecological Protection and High-Quality Development” strategy proposed in 2019 introduced stricter land-use control and restoration requirements^19^. These policy stages provide an institutional basis for interpreting the observed phased land-use transitions and ecosystem service responses.

## Research methods

**Fig. 2:**
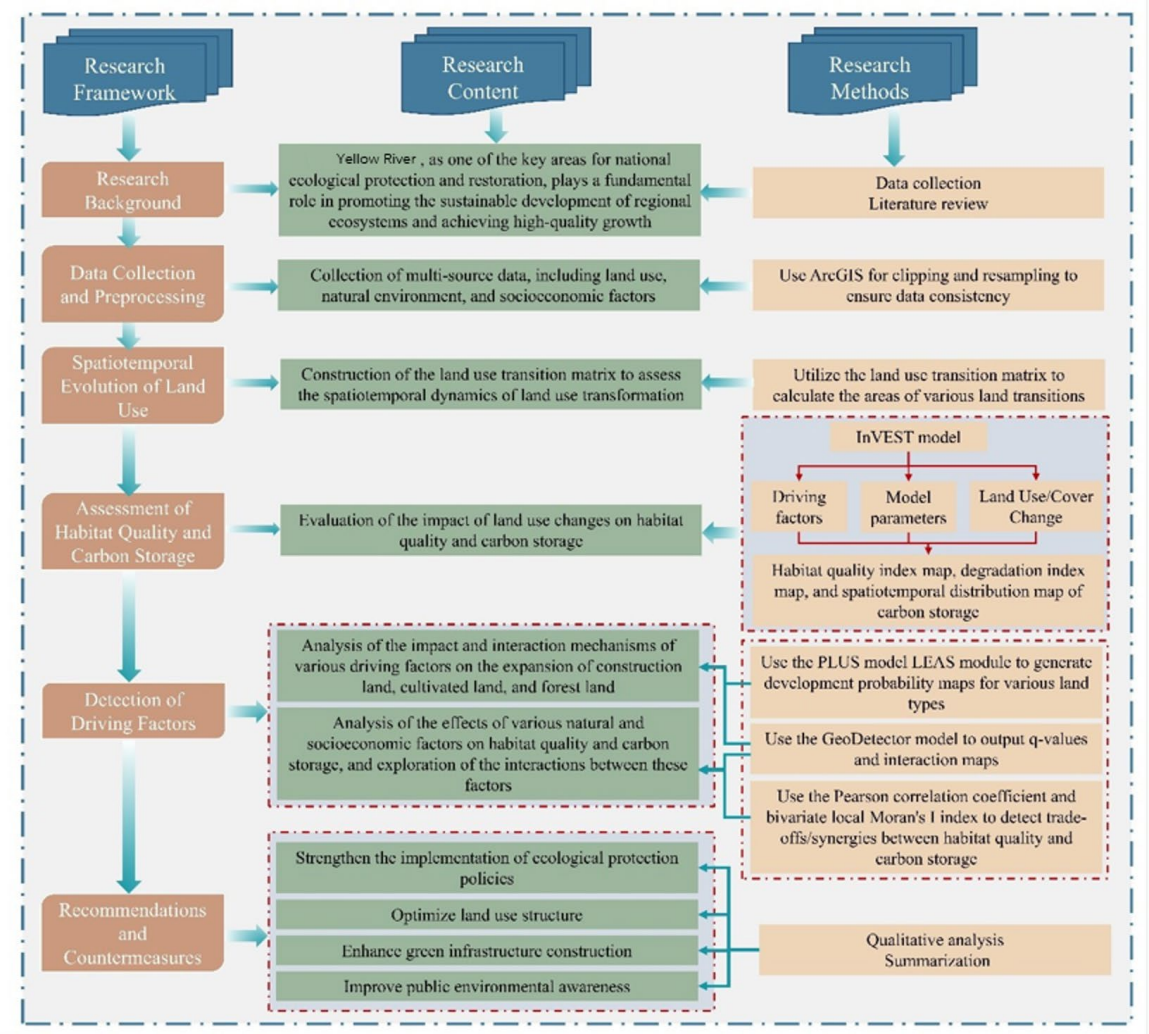
Conceptual framework.

This study designed and implemented an integrated analytical framework that combines multiple methodological approaches to systematically examine the multidimensional impacts of land-use change on water yield and carbon storage in the middle reaches of the Yellow River^20^. The research workflow involves characterizing the spatiotemporal dynamics of land use through a land-use transition matrix, simulating land-use pattern evolution using the LEAS module of the PLUS model, quantifying ecosystem services via the InVEST model, identifying driving factors with the GeoDetector method, and conducting correlation analyses, thereby elucidating the dynamic patterns of water yield and carbon storage and their key driving mechanisms. The overall research framework is illustrated in Fig. 2.

The proposed framework follows a hierarchical and sequential logic of “diagnosis–prediction–decision support” rather than a simple juxtaposition of independent models.

Specifically, the diagnostic component constitutes the foundational layer by identifying the current system state, dominant drivers, and key spatiotemporal patterns based on historical observations. The outputs of the diagnostic analysis, including parameter constraints and dominant transition characteristics, are then directly fed into the prediction component, which simulates future land-use and ecosystem service dynamics under different scenarios. Building upon the prediction results, the decision-support component represents the highest level of the framework, translating simulated outcomes into management-relevant indicators to support policy evaluation and adaptive planning.

Therefore, information flows unidirectionally from diagnosis to prediction and finally to decision support, forming an internally coupled and logically consistent modeling system.

### Land use transfer matrix

The land use transfer matrix is employed to quantify the conversion relationships and transition rates among different land use types over time, thereby revealing the spatiotemporal dynamics of land use change^21^. In this study, the transfer matrix is applied to analyze the spatial transformation processes and trends of land use types in the middle reaches of the Yellow River from 1985 to 2023. The formula is as follows:

1 $${A_{ij}}=\left[ {\begin{array}{*{20}{c}} {{A_{11}}}&{{A_{12}}}& \cdots &{{A_{1n}}} \\ {{A_{21}}}&{{A_{22}}}& \cdots &{{A_{2n}}} \\ \cdots & \cdots & \cdots & \cdots \\ {{A_{n1}}}&{{A_{n2}}}& \cdots &{{A_{nn}}} \end{array}} \right]$$

where A_ij_ represents the area transferred from land use type *i* to type *j*, and *n* denotes the total number of land use categories. Each row of the matrix corresponds to the outflow information of land type *i*, while each column reflects the inflow information of land type *j*^22^.

### InVEST model

#### Water yield module

This study employed the InVEST model to quantitatively assess ecosystem services. The model has been widely used to investigate the impacts of land-use/cover change on ecosystem functions^23^. In this study, we selected the water yield and carbon storage modules to quantitatively analyze the evolution of the ecological environment in the middle reaches of the Yellow River from 1985 to 2023.

To comprehensively assess water yield in the study area, a water yield estimation model tailored to the middle reaches of the Yellow River was systematically developed, enabling spatial quantification by integrating key climatic and ecological factors^24^. The model considers the entire input–storage–output process of the hydrological cycle, incorporating the following core variables: mean annual precipitation as the primary water input, annual potential evapotranspiration representing regional evapotranspiration capacity, vegetation-available water content reflecting the hydrological regulation function of vegetation, and soil depth-determined water storage capacity^25^. These variables, combined with biophysical parameters corresponding to different land-use types, form the computational framework of the model through spatial heterogeneity analysis and temporal dynamics extraction (Table 2). The calculation formula is as follows:

2 $$Y\left( x \right)=\left( {P\left( x \right) - AET\left( x \right)} \right) \cdot \frac{1}{A}$$

3 $$AET\left( x \right)=P\left( x \right) \cdot \frac{{PET\left( x \right)}}{{PET\left( x \right)+P\left( x \right)PET\left( x \right)}}$$

where Y(x) is the annual water yield of cell x; P(x) is the annual precipitation of cell x; AET(x) is the annual actual evapotranspiration of cell x; A is the watershed area, used to convert water depth units to volume; and PET(x) is the potential evapotranspiration of cell x. All units are in millimeters (mm)^26^.

**Table 2:** Biophysical parameters of different land use types.

Land-use type	Maximum root depth (mm)	Evapotranspiration coefficient (Kc)	LULC_veg
Cropland	2000	0.68	1
Forest	5200	0.95	1
Grassland	2400	0.65	1
Water Body	100	1	0
Built-up Land	100	0.3	0
Unused Land	300	0.2	0

Although the InVEST model provides a set of general biophysical parameters, the default values may not accurately reflect the hydro-ecological characteristics of the arid–semi-arid transition zone of the Middle Yellow River Basin. To improve model applicability, we conducted a localized adjustment of several key parameters, including plant available water content (PAWC), root depth, and evapotranspiration coefficient (Kc).

1. PAWC values were calibrated according to the soil texture dataset and published field observations in the Loess Plateau, and were reduced from the default 0.15–0.20 to 0.06–0.08 for sandy–loam soils.
2. Maximum root depth ranges of major vegetation types were modified following regional ecological studies.
3. Kc values for cropland and grassland were adjusted within the lower bounds of the ranges reported for semi-arid regions.

These localized adjustments effectively reduce uncertainties caused by mismatches between default parameters and local hydro-ecological conditions.

#### Carbon storage module

The carbon storage module is used to estimate the carbon stocks of different land-use types and is divided into four carbon pools: aboveground biomass, belowground biomass, soil carbon, and dead organic matter^27^. The total carbon storage is calculated as follows:

4 $${C_{total}}={C_{above}}+{C_{below}}+{C_{soil}}+{C_{dead}}$$

where C_total_ is the total carbon storage in Mg/ha, and C_above_, C_below_, C_soil_, and C_dead_ represent the carbon stocks in aboveground biomass, belowground biomass, soil, and dead organic matter, respectively (units: Mg/ha)^28^.

In the InVEST carbon storage module, carbon is allocated to different land-use types (e.g., forest, grassland, cropland) based on land-use data and carbon pool density tables (see Table 3)^29^. Carbon pool density parameters were set according to the actual ecological conditions in the middle reaches of the Yellow River, with reference to relevant literature and the InVEST User Guide. The output of this module reflects both the distribution and total amount of carbon storage under different land-use types, providing a scientific basis for quantifying the impacts of land-use change on carbon stocks^30^.

**Table 3:** Carbon density of different land use types and their components.

Land use types	C_above	C_below	C_soil	C_dead
Nodate	0	0	0	0
Cropland	4.85	0.92	58.2	2.84
Forest	20.92	7.53	67.3	54.2
Grassland	1.63	8.48	60.2	2.19
Water	0	0	62.1	0
Impervious	0	0	60	0
Barren	0	0	53.3	0

The carbon density parameters for the four carbon pools (Table 3) were compiled and synthesized from a substantial body of peer-reviewed literature focusing on the Loess Plateau and similar ecological zones. For instance, the carbon density for forest ecosystems accounts for the dominance of secondary growth and plantation forests in the region, which typically have lower biomass carbon compared to mature natural forests. The soil carbon values were calibrated to reflect the generally moderate to low organic carbon content of Loess soils, while considering the enhancing effect of long-term conservation practices. This region-specific parameterization enhances the reliability of our carbon storage estimates.

### PLUS model

The PLUS (Patch-generating Land Use Simulation) model integrates the Random Forest algorithm with Cellular Automata (CA) technology, enabling dynamic simulation of land-use change^31^. This model can capture transitions between land-use types while reflecting the spatial patterns of land-use change^32^.

- Calculation of land-use development probability using random forest

The Random Forest algorithm evaluates the contribution of each driving factor to land-use type transitions through an ensemble of decision trees, ultimately generating the development probability for each land-use type^33^. The development probability is calculated as follows:

5 $$P_{{RF}}^{k}\left( {xi} \right))=\frac{1}{M}\sum\limits_{{m=1}}^{M} I \left( {{h_m}\left( {{x_{i}}} \right)=k} \right)$$

where *M* is the number of decision trees, *h*_*m*_(*x*_*i*_) is the prediction result of the *m* tree, and *I* is the indicator function^34^.

b. Cellular automata transition rules

The Cellular Automata (CA) adjust land-use type transitions dynamically based on the states of neighboring cells and local rules^35^. The transition rules incorporate an adaptive inertia coefficient to govern the smoothness of land-use changes:

6 $${T_{i \to k}}=P_{{RF}}^{k}\left( {{x_i}} \right) \times {\Omega _{adaptive}} \times \left( {1+\gamma \times rand(0,1)} \right)$$

where *Ω*_*adaptive*_ represents the adaptive inertia coefficient and *γ* denotes the stochastic disturbance factor^36^.

c. Patch-generating mechanism

In the PLUS model, patch generation is based on a threshold-decreasing strategy, which dynamically adjusts the conversion-area threshold to achieve the spatial expansion of land-use types^37^. The core formula of the patch-generating mechanism is as follows:

7 $${A_{patch}} \geqslant \theta \times {A_{total}}$$

In the equation, *A*_*patch*_ denotes the area of the candidate patch, $\theta$ represents the decreasing threshold (with an initial value of 0.3 and a step size of 0.01), and *A*_*total*_ signifies the total demand area^38^.

d. Validation of PLUS simulation accuracy

To evaluate the reliability of the land-use simulation results, we conducted an accuracy assessment by comparing the PLUS-simulated land-use map with the actual land-use data for the corresponding historical year. The validation metrics included the overall accuracy (OA) and Kappa coefficient, which are widely used to quantify the agreement between model simulations and observed land-use patterns.

The confusion matrix was generated by overlaying the simulated and observed maps, and the Kappa coefficient was calculated as:

8 $$Kappa=\frac{{Po - Pe}}{{1 - Pe}}$$

where ${P}_{o}$ is the observed agreement and ${P}_{e}$ is the agreement expected by chance. A Kappa coefficient above 0.75 is generally considered to indicate good simulation performance.

In this study, the PLUS model achieved an overall accuracy of 84.6% and a Kappa coefficient of 0.79, demonstrating that the simulated land-use pattern is consistent with historical observations and can be reliably used for subsequent ecosystem service assessments.

### GeoDetector model

The GeoDetector framework provides a quantitative means of evaluating spatial heterogeneity, offering insights into the extent to which both natural conditions and socio-economic variables affect ecosystem service changes^39^. In this study, the GeoDetector model was applied to quantify the impacts of various natural and socioeconomic factors on the changes of four ecosystem services in the middle reaches of the Yellow River^40^. By analyzing the spatial heterogeneity of these services, the model helps reveal the combined effects of climate change and human activities^41^. The model outputs the explanatory power of each driving factor, with higher values indicating a greater influence on the variation of ecosystem service functions. The significance of the q-statistic for each factor and interaction was assessed using a permutation test (1000 permutations) at a confidence level of *p* < 0.05. The calculation formula of the model is as follows:

9 $$q=1 - \frac{{\sum\nolimits_{{h=1}}^{L} {{N_h}\sigma _{h}^{2}} }}{{N{\sigma ^2}}}=1 - \frac{{SSW}}{{SST}}$$

10 $$SSW=\sum\limits_{{h=1}}^{L} {{N_h}\sigma _{h}^{2},SST=N{\sigma ^2}}$$

Among them, q∈[0,1], where a larger value indicates a stronger explanatory power of the independent variable on water yield. *SSW* and *SST* represent the within-stratum variance and the total variance of the entire region, respectively. *Nh* and *NN* are the number of units in stratum h and the entire region, respectively. *σ*_*h*_^*2*^ and *σ*^*2*^ denote the variance of stratum *h* and *γ* the entire region, respectively^42^.

### Correlation and spatial autocorrelation analysis

This study employed the Pearson correlation coefficient and bivariate local Moran’s I to quantitatively explore the relationship between water yield and carbon storage, as well as their spatial clustering and differentiation characteristics^43^. The statistical significance of the Pearson correlation coefficient (r) and both global and local Moran’s I was tested. A result was considered statistically significant if its p-value was less than 0.05.

#### Correlation analysis

The correlation analysis applied the Pearson correlation coefficient to determine the linear linkage between water yield and carbon storage^44^. This coefficient functions as a statistical metric describing associations between two datasets, and its expression is shown as follows:

11 $$r=\frac{{\sum\nolimits_{{i=1}}^{n} {\left( {{x_i} - \bar {x}} \right)\left( {{y_i} - \bar {y}} \right)} }}{{\sqrt {\sum\nolimits_{{i=1}}^{n} {{{\left( {{x_i} - \bar {x}} \right)}^2}} } .\sqrt {\sum\nolimits_{{i=1}}^{n} {{{\left( {{y_i} - \bar {y}} \right)}^2}} } }}$$

In the formula, *r* denotes the Pearson correlation coefficient; *x*_*i*_ and *y*_*i*_ represent the *i* data points of the two variables, respectively; *x* and *y* signify the mean values of the two variables; and *n* corresponds to the sample size.

#### Bivariate local Moran’s I

To further analyze the spatial distribution patterns of water yield and carbon storage, a bivariate local spatial autocorrelation method was employed. This approach uses the bivariate local Moran’s I to reveal the spatial clustering and differentiation patterns between the two ecosystem service variables. The calculation formula is as follows:

12 $$I_{i} = \frac{{n\left( {x_{i} - \bar{x}} \right)\sum\nolimits_{{j = 1}}^{{\mathrm{n}}} {W_{{ij}} \left( {x_{j} - \bar{x}} \right)} }}{{\sum\nolimits_{{i = 1}}^{n} {\left( {x_{i} - \bar{x}} \right)^{2} } }}$$

In the formula, *I*_*i*_ denotes the Local Moran’s I; *n* is the total number of spatial units; *x*_*i*_ and *x*_*j*_ represent the ecosystem service values of spatial units *i* and *j*, respectively; x is the mean of the observed values; and $\omega$_*ij*_ constitutes the spatial weight.

### Selection of driving factors

To comprehensively investigate the integrated effects of land-use transitions on water yield and carbon storage, this study selected natural and socioeconomic driving factors closely related to ecosystem services. The selection of driving factors was informed by the geographical characteristics, ecological attributes, and socioeconomic conditions of the middle reaches of the Yellow River.

#### Natural driving factors

**Fig. 3:**
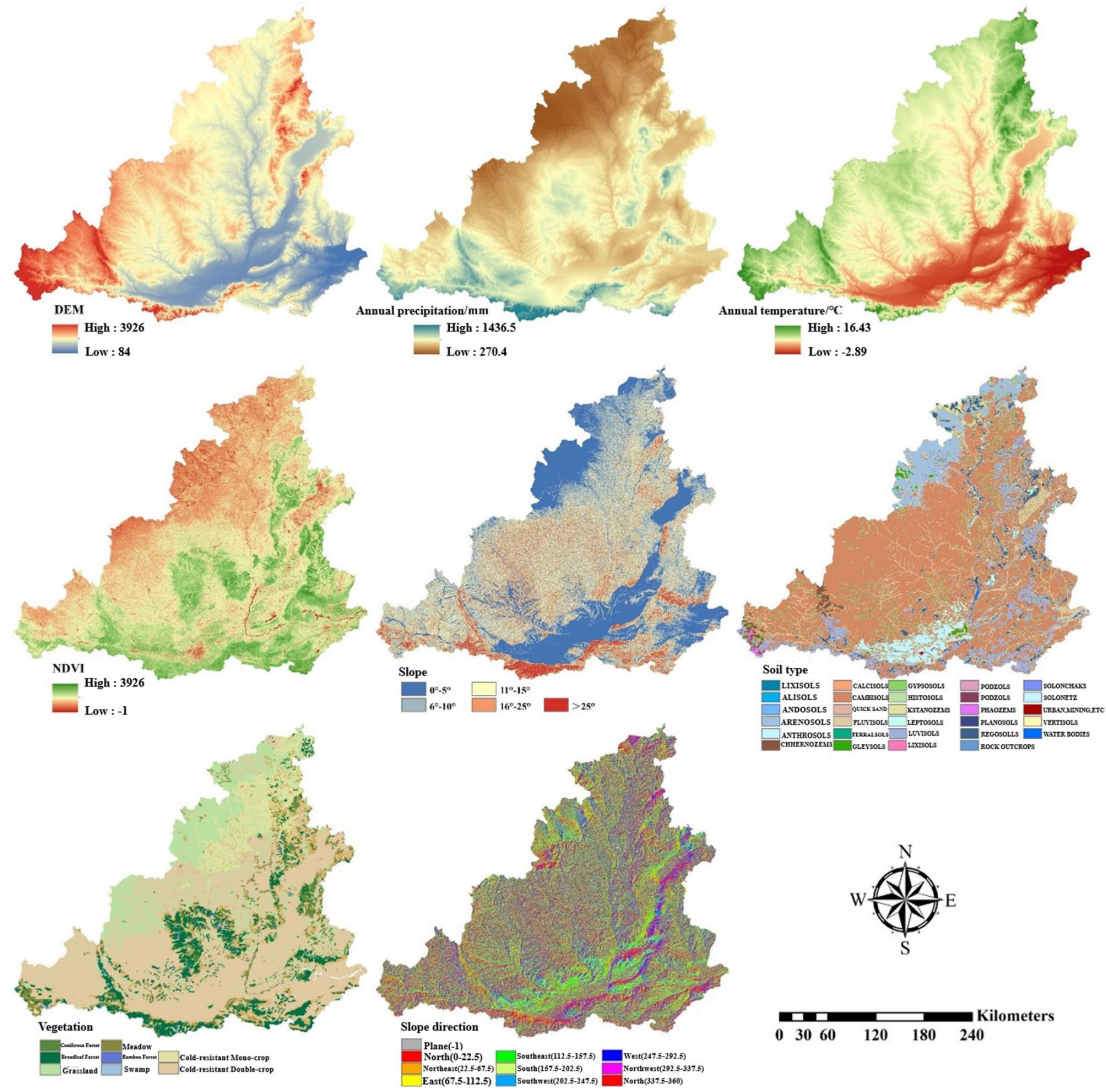
Spatial visualization of natural factor driving factors These maps were created using ArcGIS Pro 3.4 (https://www.esri.com/software/arcgis-pro). The spatial data sources are detailed in Table 1.

Natural factors constitute the fundamental conditions influencing ecosystem services. To comprehensively assess their impact on land-use change, eight key natural driving factors were selected (Fig. 3). As critical drivers of climate change, annual precipitation and mean annual temperature affect regional ecological stability by regulating plant growth, ecosystem productivity, and service functions. Climate factors primarily influence photosynthetic efficiency, evapotranspiration processes, and carbon cycling, thereby modulating the supply capacity of ecosystem services. The subtropical–temperate transitional characteristics of the middle reaches of the Yellow River provide an ideal natural laboratory for such studies. Topographic factors (DEM, slope, and aspect) shape land-use patterns (e.g., flat areas favor agriculture, steep slopes favor forestry or orchards), soil spatial heterogeneity, and hydrological processes, thus determining the spatial distribution of ecosystem services. The region’s diverse soil types not only support crop production but also play a key role in nutrient cycling and carbon sequestration, with their heterogeneity directly affecting agricultural output, ecosystem service supply, and system stability. Additionally, vegetation type and its proxy indicator NDVI, as comprehensive metrics of ecosystem health, reflect both dynamic vegetation coverage and indirectly assess service provision capacity. Changes in NDVI are closely related to habitat quality and the evolution of ecosystem service functions.

#### Socioeconomic driving factors

**Fig. 4:**
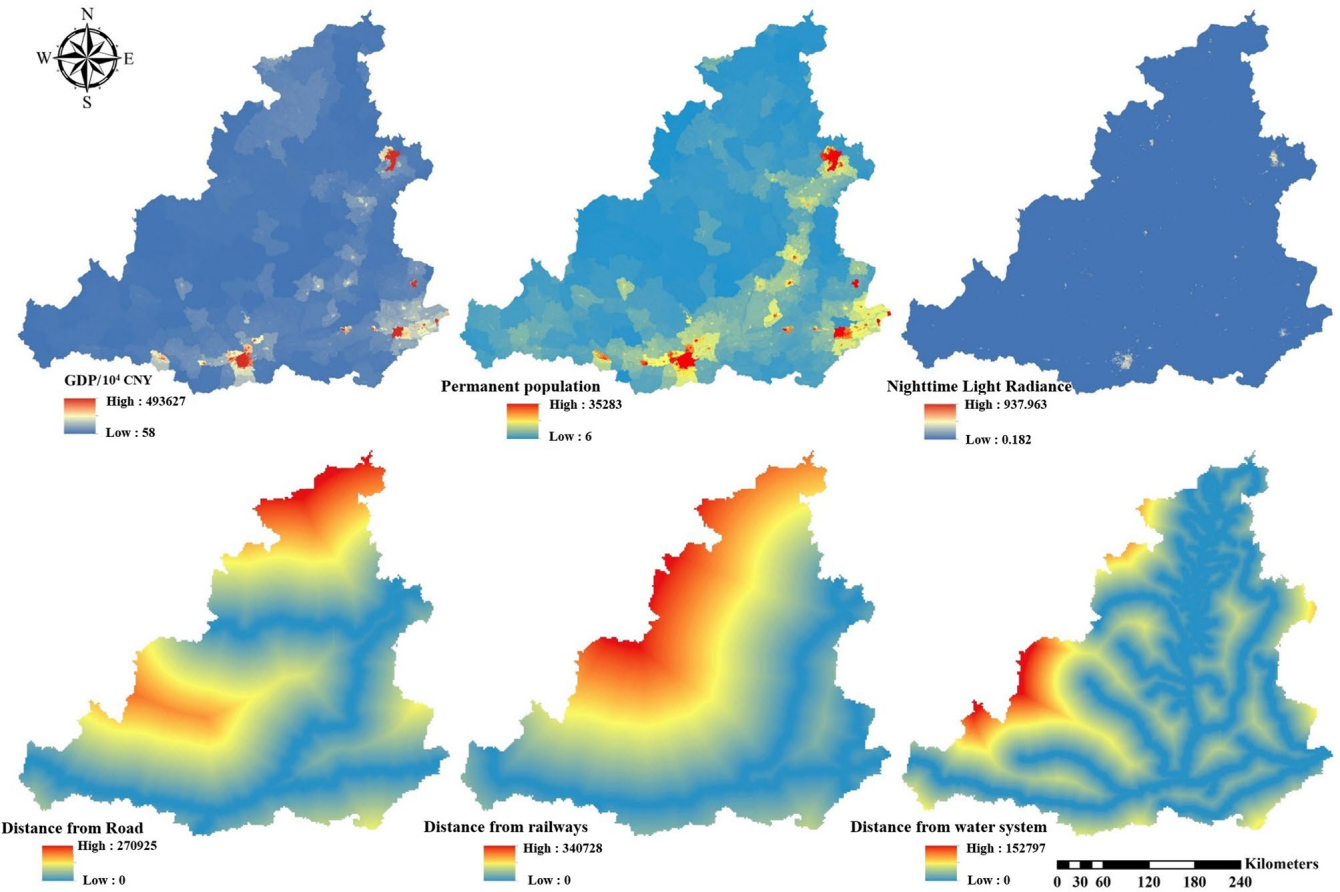
Spatial visualization of socio-economic factor driving factors These maps were created using ArcGIS Pro 3.4 (https://www.esri.com/software/arcgis-pro). The spatial data sources are detailed in Table 1.

Socioeconomic factors, as external drivers, profoundly influence land-use patterns and ecological changes. In this study, six key socioeconomic driving factors were selected (Fig. 4).

Population distribution, as an important indicator of human activity intensity, exhibits significant spatial clustering, which intensifies land-use pressure. This phenomenon is particularly pronounced in rapidly urbanizing areas of the middle reaches of the Yellow River, directly contributing to increased ecological stress. Gross Domestic Product (GDP), as a core measure of regional economic development, not only reflects the scale of the economy but also affects land-use patterns and ecosystem service demands through changes in industrial structure and spatial layout. In regions experiencing rapid economic growth, the coordinated advancement of industrialization and urbanization drives fundamental shifts in land-use types and triggers systemic changes in ecosystem service demand. The nighttime light index, as a novel indicator representing the spatialization of economic activities, leverages high-resolution remote sensing data to accurately capture the intensity and spatial distribution patterns of regional economic activity. Moreover, infrastructure accessibility, represented by the distribution of transportation networks and water systems, not only directly influences land development potential but also guides land-use decisions by altering locational advantages.

It is recognized that certain socioeconomic driving factors, such as GDP, population density, and the nighttime light index, are often correlated as they collectively reflect the intensity of human activities and economic development. However, a key advantage of the GeoDetector model is that it is based on spatial stratification heterogeneity, which is immune to the multicollinearity issues that often plague traditional regression-based models. This is because GeoDetector assesses the explanatory power of each factor by examining the similarity within categories and the differences between them, without relying on the covariance structure between independent variables.

To further ensure the robustness of our factor selection, we calculated the Pearson correlation coefficients between these factors. While moderate to high correlations were observed (e.g., between GDP and nighttime light index, *r* > 0.7), the GeoDetector’s factor detection and interaction detection results still effectively revealed the unique and interactive contributions of each factor to the spatial heterogeneity of ecosystem services, validating their inclusion in our analysis.

## Spatiotemporal dynamics and driving mechanisms

### Spatiotemporal evolution of land use

**Fig. 5:**
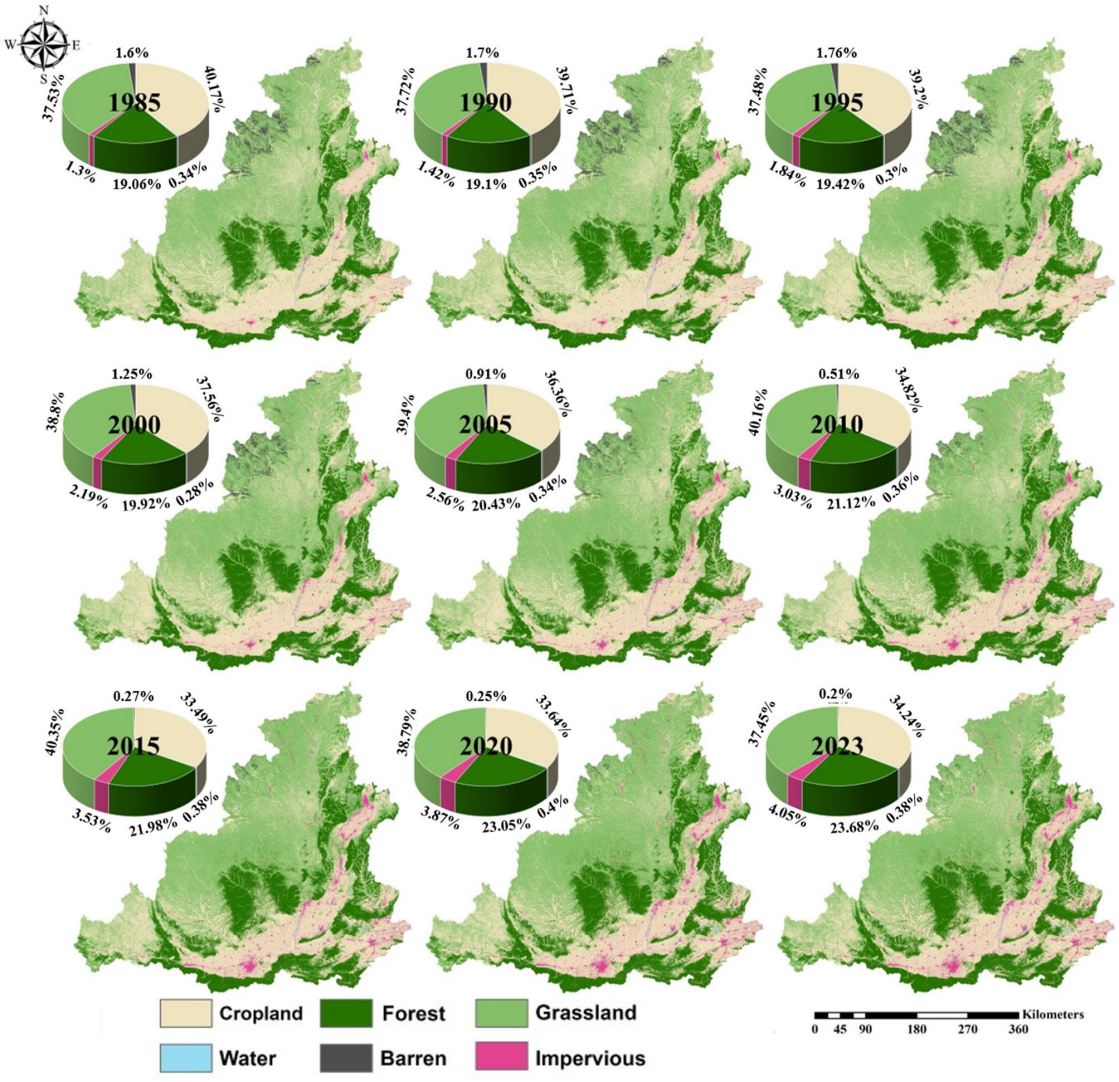
Land use type distribution map of the study area from 1985 to 2023 These maps were created using ArcGIS Pro 3.4 (https://www.esri.com/software/arcgis-pro). The land use data were obtained from the Resource and Environmental Science and Data Center (https://www.resdc.cn).

Based on land-use distribution data for the middle reaches of the Yellow River from 1985 to 2023 (Fig. 5), the main land-use types in the study area include cropland, forest, grassland, built-up land, water bodies, and unused land. Over the past four decades, significant changes in land-use patterns have occurred, driven by both natural and anthropogenic factors. Overall, the area of cropland has shown a continuous decline, while forest and built-up land have increased significantly. Changes in grassland and unused land, however, exhibit notable regional heterogeneity. Natural conditions constrain the spatial potential for land-use conversion, whereas policy orientation, urbanization, and socioeconomic development have served as dominant driving forces.

**Fig. 6:**
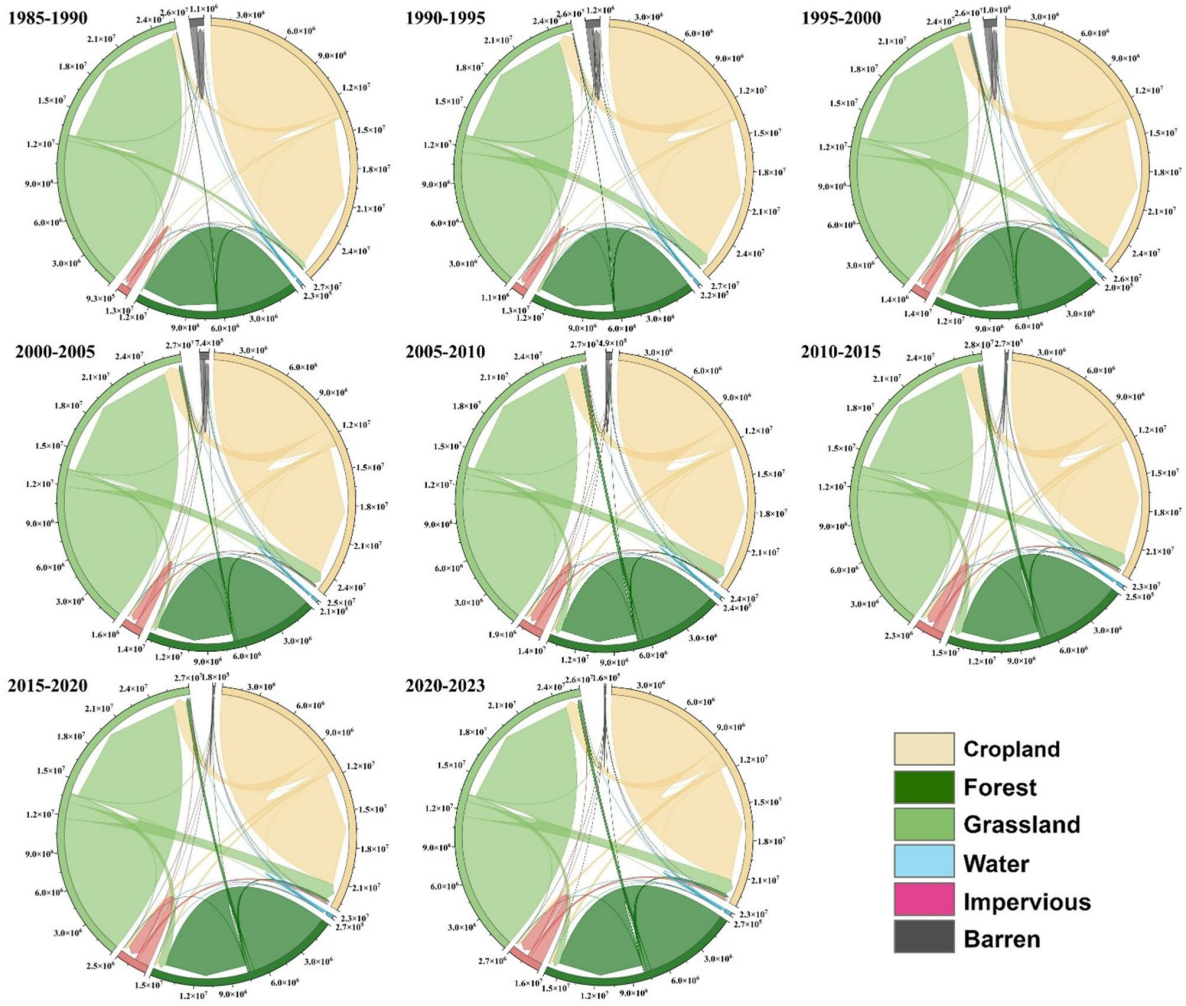
Land use type transfer chord diagram of the study area from 1985 to 2023.

The land-use transition matrices (Tables 4, and Appendix: 6, 7, 8, 9, 10, 11, 12, 13) and chord diagrams (Fig. 6) visually illustrate the dynamic change patterns of land-use types between 1985 and 2023. From 1985 to 1990, the total land transitioned amounted to 1.3188 million ha (3.84% of the study area). This increased to 3.1850 million ha (9.27%) during 1990–1995, 3.8439 million ha (11.18%) during 1995–2000, 3.7559 million ha (10.93%) during 2000–2005, 4.6659 million ha (13.58%) during 2005–2010, and reached its peak of 4.9195 million ha (14.31%) during 2010–2015. It then slightly declined to 4.6050 million ha (13.40%) in 2015–2020 and 4.4687 million ha (13.00%) in 2020–2023.The spatiotemporal characteristics of land-use evolution varied across different periods. Between 1985 and 2000, Agricultural Expansion and Economic Development. This period was primarily characterized by rapid cropland expansion to meet the demands of population growth and national food security strategies, leading to a net decrease in forest and grassland areas. From 2000 to 2010, the study area entered the onset phase of large-scale ecological restoration driven by national policies. The Grain-for-Green Program (GFGP) was officially launched nationwide in 1999 and rapidly expanded during 2000–2005, with the middle reaches of the Yellow River identified as one of the key implementation regions. According to the Ministry of Natural Resources and previous studies, more than 3.2 × 10⁶ ha of sloping cropland (slope > 25°) in the Loess Plateau were converted into forest and grassland during this period, with annual investment intensity and implementation strength peaking between 2002 and 2006^45^. Meanwhile, the Three-North Shelter Forest Program entered its fourth phase (2001–2010), further promoting vegetation restoration at the basin scale. These policy-driven land conversions directly reshaped land-use structure and constituted the dominant driver of forest and grassland expansion in this decade. This policy-driven shift resulted in a significant net increase in forest area and the partial recovery of grassland during this decade, even as built-up land expansion continued. Between 2010 and 2023, Ecological Conservation under Accelerated Urbanization. This period saw the continued enforcement and maturation of earlier ecological projects. Furthermore, it encompasses the era of China’s “Ecological Civilization” construction, formally elevated to a national strategy in 2012, and the later proposal of the “Yellow River Basin Ecological Protection and High-Quality Development” strategy in 2019. These high-level directives reinforced vegetation restoration efforts. However, simultaneously, urbanization accelerated markedly, creating intense competition for land and exerting greater pressure on remaining cropland and ecological spaces.

**Table 4:** Summary of major land use transfers and dynamics (1985–2023).

Period	Total transferred area (hm²)	% of study area	Key land use change trends	
			Main	Minor
1985–1990	1,318,800	3.84	Cropland → grassland	Cropland → forest
1990–1995	3,185,000	9.27	Grassland → cropland	Cropland → grassland
1995–2000	3,843,900	11.18	Cropland → grassland	Grassland → cropland
2000–2005	3,755,900	10.93	Cropland → grassland	Grassland → cropland
2005–2010	4,665,900	13.58	Cropland → built-up	Grassland → cropland
2010–2015	4,919,500	14.31	Cropland → built-up	Grassland → cropland
2015–2020	4,605,000	13.40	Forest → cropland	Grassland → cropland
2020–2023	4,468,700	13.00	Cropland → forest	Cropland → built-up

The complete transition matrices for all periods are provided in Appendix: Tables 6, 7, 8, 9, 10, 11, 12, 13.

Land use change in the study area is strongly influenced by multiple socio-economic factors. During 1985–2023, rapid population growth and continuous urban expansion significantly accelerated the conversion of cultivated land and unused land into construction land. The increase in regional GDP and industrial restructuring also promoted land development, particularly in the central and downstream plains where transportation and economic activities are concentrated. In contrast, ecological restoration programs—such as the Grain-for-Green Project and soil conservation policies—contributed to the expansion of forest and grassland areas in the upstream and hilly regions. These socio-economic forces collectively shaped the spatial and temporal land use trajectories observed in the study area.

From the perspective of spatial heterogeneity, land-use changes exhibit significant regional differences. In the mid- and upper reaches (e.g., the southern Taihang Mountains), forest and grassland areas increased, reflecting the effectiveness of ecological restoration projects. In the mid- and lower reaches, cropland reduction and built-up land expansion were predominant, driven by urbanization and industrialization. Ecologically fragile areas (e.g., northern desert margins) faced grassland degradation. Land-use changes have profound implications for ecosystem service functions. On one hand, the expansion of forest and grassland enhances water conservation, soil retention, and biodiversity protection. On the other hand, cropland loss and built-up land expansion may threaten food security and ecosystem stability, especially in rapidly urbanizing areas, where intensive land development further exacerbates ecosystem pressure. Therefore, future land-use planning should prioritize the coordinated development of ecosystem service functions to promote regional sustainability. The driving effects of socio-economic factors exhibited clear spatial heterogeneity across the study area. Urbanization and economic development played dominant roles in the middle and lower reaches, where cities and transportation networks are dense, resulting in significant growth of built-up land and reduction of cultivated land. However, in the upper reaches and ecologically fragile zones, ecological restoration policies were the primary drivers of land use change, leading to substantial increases in forest and grassland. These regional differences indicate that land use dynamics were not governed by a single factor but by varying socio-economic and policy drivers in different geographic settings.

### Spatiotemporal evolution of water yield

**Fig. 7:**
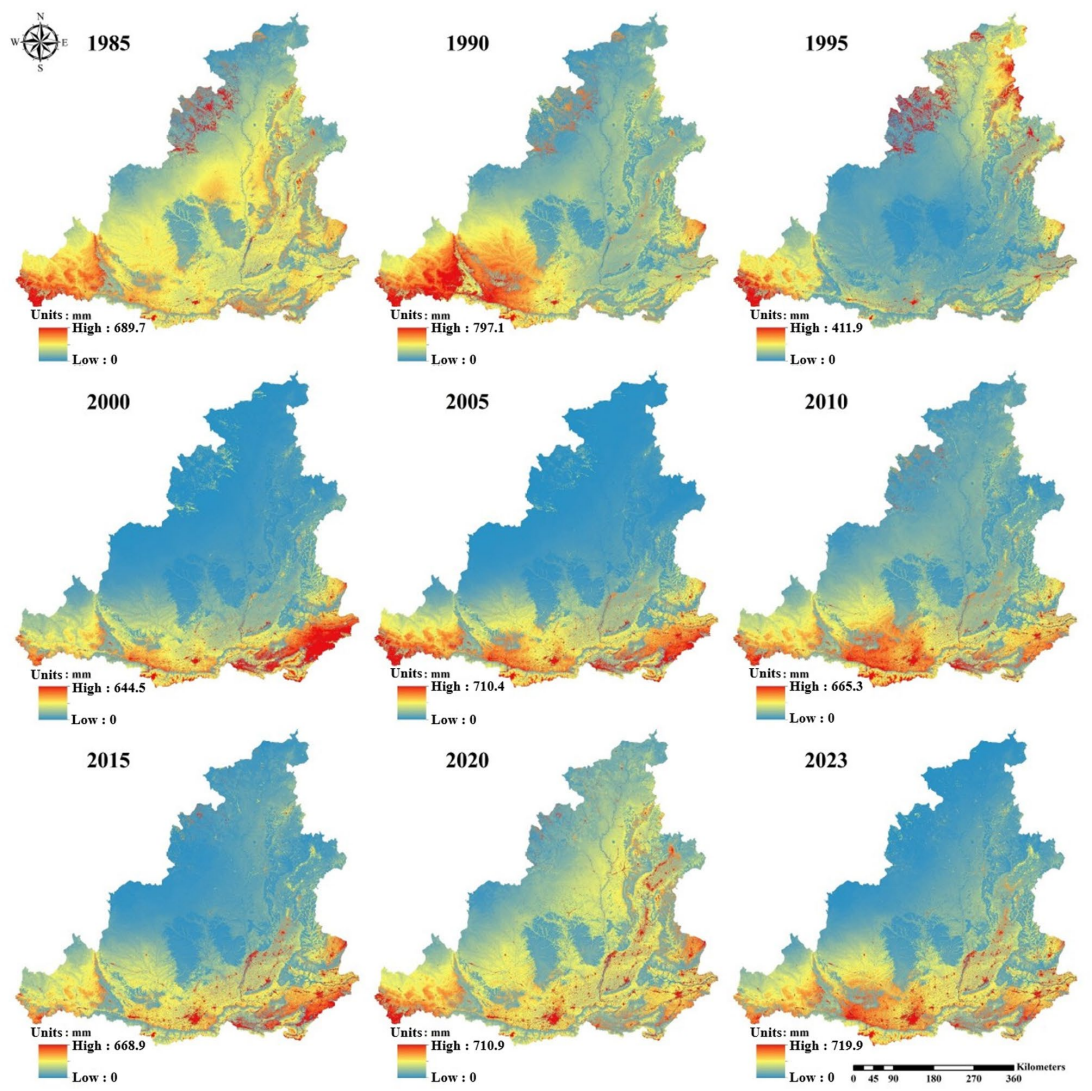
Spatiotemporal distribution map of water yield in the study area from 1985 to 2023 These maps were created using ArcGIS Pro 3.4 (https://www.esri.com/software/arcgis-pro). The spatial data sources are detailed in Table 1.

The InVEST model results for water yield in the middle reaches of the Yellow River from 1985 to 2023 (Fig. 7) show that the spatial distribution exhibits a “nested double-peak” pattern. The primary peak is located in the central-southern Fenwei Plain and the southeastern piedmont of the Taihang Mountains, while the secondary peak occurs along the southeastern edge of the Ordos Plateau. In contrast, the northern Yulin–Yan’an arid zone and the northwestern Mu Us Desert consistently record water yields below 200 mm, forming distinct low-value areas.

Temporal analysis indicates that water yield experienced phased fluctuations during 1985–2023. Between 1985 and 1995, water yield declined sharply, closely linked to intensified human activities and changes in land-use structure. From 1995 to 2010, water yield in the primary peak area recovered to 655.3 mm by 2010, representing an 82.7% increase, primarily benefiting from the implementation of the Three-North Shelter Forest Program and the promotion of water-saving irrigation technologies. In the secondary peak area, water yield fluctuated downward by 12.3% due to coal mining activities, creating an “ecology–economy” conflict zone. During 2010–2023, water yield in the primary peak area stabilized at 710.9 mm in 2020, reflecting a 9.1% increase relative to 1995 and demonstrating the long-term effectiveness of ecological engineering. In the secondary peak area, water yield recovered to 655.3 mm through ecological restoration of mining areas, reducing the gap with the primary peak region. Furthermore, pixel-level analysis of average water yield shows that although the overall trend has improved, regional differences remain significant. Along the main course of the Yellow River and at the edges of mountainous areas, a “high–low–high” pattern emerges, primarily driven by variations in groundwater recharge and slope runoff regulation.

### Spatiotemporal evolution of carbon storage

**Fig. 8:**
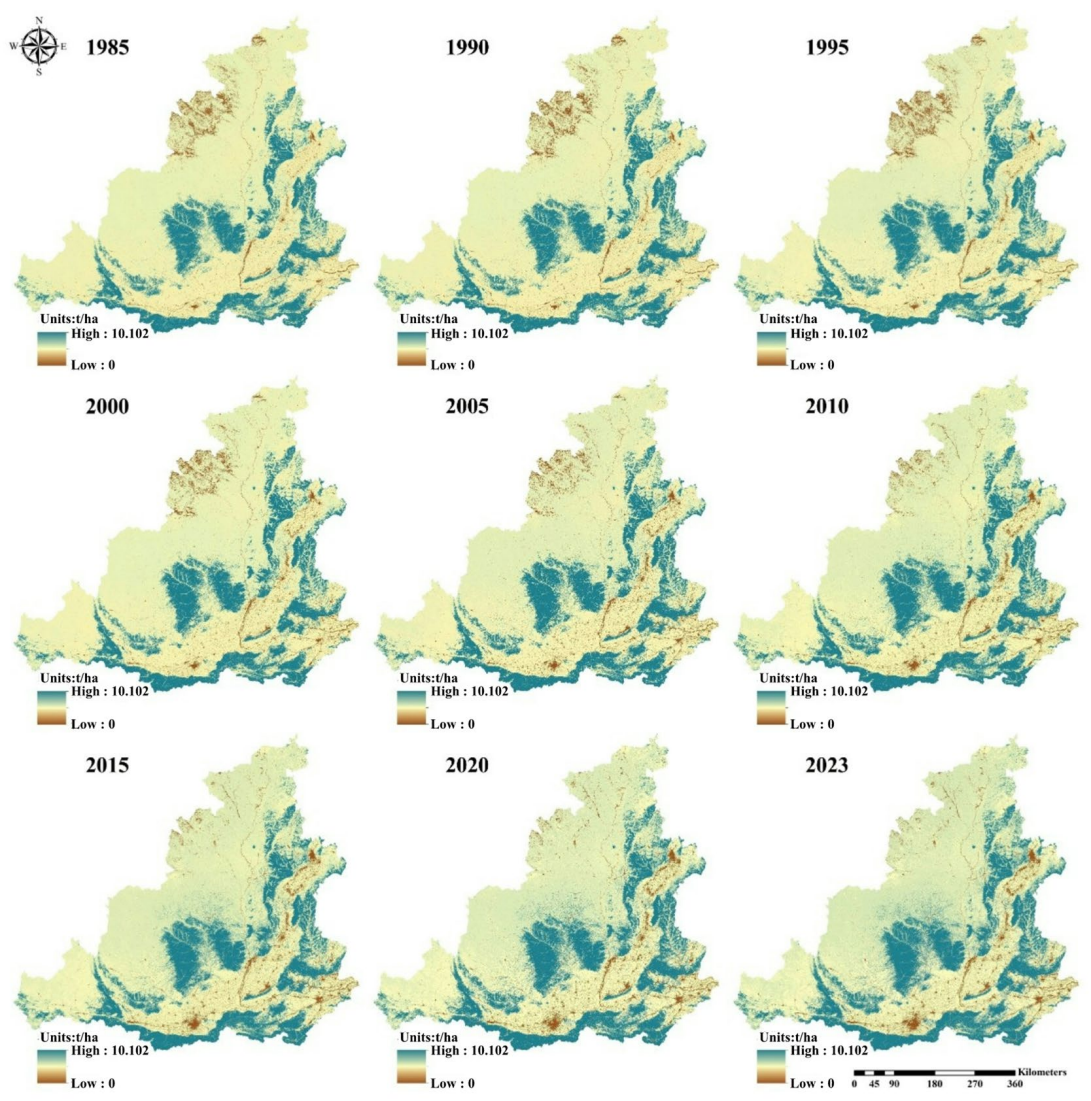
Spatiotemporal distribution map of carbon storage in the study area from 1985 to 2023 These maps were created using ArcGIS Pro 3.4 (https://www.esri.com/software/arcgis-pro). The spatial data sources are detailed in Table 1.

From 1985 to 2023, carbon storage in the middle reaches of the Yellow River exhibited significant spatiotemporal heterogeneity (Fig. 8). The spatial pattern evolved through three distinct stages: “stable ecological barrier zones—expansion of low-value areas—isolation of urban clusters.” High-value areas (> 10 t/ha) in the Taihang Mountains and southern Lüliang Mountains remained stable, reflecting a “topography–vegetation synergy” shaped by terrain and precipitation distribution. Low-value areas (< 2 t/ha) in the Weihe–Fenhe Plain gradually expanded from west to east and from north to south, with expansion pathways negatively correlated with the growth of built-up land and the intensification of agriculture. In eastern urban clusters, three-dimensional greening measures created “carbon storage growth poles” (> 4 t/ha).

Temporal analysis reveals a dual “policy regulation–natural response” driving mechanism. During 1985–2000, the pilot phase of the “Grain-for-Green” program, carbon storage in the northern mountainous areas recovered and was significantly higher than in the central plains. From 2000 to 2023, under intensified policy enforcement, carbon storage increased substantially in ecological protection areas, while urbanized regions experienced accelerated decline. Carbon trading pilots (e.g., in Ordos) promoted emissions reduction in the central plains, but the expansion of built-up land reduced ecological land, resulting in a complex pattern of coexisting “carbon storage islands” and “low-carbon corridors.” Furthermore, ecological restoration projects significantly enhanced local carbon storage. Vegetation improvements in mountainous and protected areas drove growth, effectively mitigating the negative impacts of urbanization.

### Trends in ecosystem service functions

Table 5 summarizes the evolution of ecosystem service functions in the study area from 1985 to 2023. Water yield exhibited a “fluctuation–recovery” pattern. Between 1985 and 1995, extreme droughts and land-use expansion caused water yield to drop sharply from 35.582 × 10⁹ m³ to 11.459 × 10⁹ m³, a decline of 68%. After 2000, the implementation of water-saving technologies and hydraulic projects led to a gradual recovery, reaching 32.455 × 10⁹ m³ by 2020, representing a 264% increase relative to 1995. However, in 2023, due to climate change (12% reduction in precipitation) and over-extraction of groundwater, water yield decreased again to 23.577 × 10⁹ m³, highlighting long-term challenges for water resource management. Carbon storage growth exhibited phased characteristics. Between 1985 and 2010, the annual growth rate averaged 2.1%, mainly driven by vegetation restoration. After 2010, the growth rate slowed to 0.8%, reflecting diminishing marginal returns from ecological protection. However, post-2020, the growth rate rebounded to 1.2%, largely driven by carbon trading pilot programs and the promotion of renewable energy policies.

**Table 5:** Ecosystem service function quantity in the study area from 1985 to 2023.

Year	Water yield (10nnnn^9^ m³)	Carbon storage (10⁶ t)	Pearson *r* (WY vs. CS)	*p*-value
1985	35.582	2481.03	− 0.079	n.s.
1990	33.069	2478.18	− 0.092	n.s.
1995	11.459	2474.22	− 0.21	< 0.05
2000	14.739	2488.64	− 0.19	< 0.01
2005	16.618	2496.58	− 0.21	< 0.008
2010	23.091	2507.39	− 0.23	< 0.007
2015	14.769	2517.03	− 0.34	< 0.005
2020	32.455	2524.36	− 0.29	< 0.003
2023	23.577	2528.69	− 0.26	< 0.001

*n.s.* not significant.

The spatial differentiation of ecosystem services in the study area is closely related to regional hydrological and geological conditions. Areas with higher elevation and fractured bedrock in the upper and middle reaches support stronger water conservation capacity due to greater precipitation infiltration, deeper soil profiles, and extensive vegetation cover. In contrast, the downstream plains with shallow groundwater tables and fine-textured soils exhibit relatively low water retention and soil conservation capacity.

Furthermore, regions with steep slopes and highly erodible soils show significantly higher soil conservation values, consistent with the spatial pattern of erosion control driven by topography. Areas with sparse vegetation and limited soil moisture, mostly located in the semi-arid transition zones, correspond to low water yield and habitat quality. These spatial patterns indicate that hydrological processes, geological substrate, and soil–vegetation interactions jointly shape the spatial heterogeneity of ecosystem service supply.

### Analysis of driving mechanisms

#### Main driving factors of water yield

**Fig. 9:**
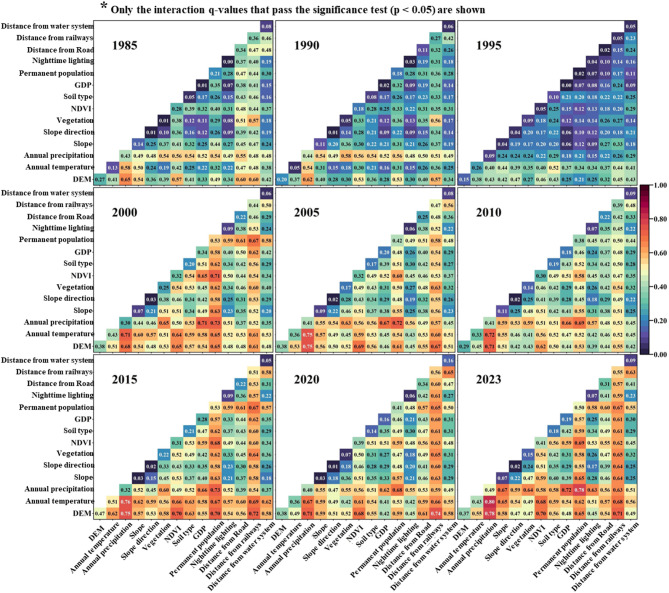
Interaction detection diagram of water yield driving factors.

Figure 9 illustrates the spatiotemporal differentiation of water yield driving factors in the middle reaches of the Yellow River from 1985 to 2023. Interactions among driving factors are mainly characterized by nonlinear enhancement and bivariate enhancement, with nonlinear enhancement accounting for 48.35–74.72%. This indicates that interaction q-values are generally higher than the independent effects of single factors, reflecting that water yield changes are driven by the synergistic effects of multiple factors. From 1985 to 2000, water yield was predominantly influenced by interactions among natural factors. Typical combinations included DEM ∩ annual precipitation (q = 0.62–0.68), DEM ∩ distance to roads (q = 0.60), and DEM ∩ distance to railways (q = 0.45–0.60), highlighting the dominant role of topography and precipitation. After 2000, socioeconomic factors became increasingly influential. Interactions such as annual precipitation ∩ population (q = 0.73,) and annual precipitation ∩ GDP (q = 0.71) indicate that economic development and population growth altered the regional water supply-demand pattern.

From 2005 to 2023, the coupling effects of natural and socioeconomic factors strengthened significantly. In 2005, combinations such as DEM ∩ annual precipitation (q = 0.75), mean annual temperature ∩ annual precipitation (q = 0.75), and annual precipitation ∩ population (q = 0.72) demonstrate how topography modulates precipitation effects on water yield, while population growth and economic activity influence its spatial distribution. Between 2010 and 2020, q-values of DEM ∩ NDVI (q = 0.62–0.70), DEM ∩ population (q = 0.70), and DEM ∩ distance to railways (q = 0.72–0.74) gradually increased, indicating that urban expansion and infrastructure development profoundly altered regional hydrological processes and vegetation coverage. By 2023, interactions such as mean annual temperature ∩ annual precipitation (q = 0.80), DEM ∩ annual precipitation (q = 0.78), and annual precipitation ∩ population (q = 0.78) were highly significant, further confirming that climate change, population pressure, and socioeconomic development jointly drive the spatiotemporal evolution of water yield. Overall, changes in water yield are the result of multi-scale interactions between natural and social systems. Future water resource management should strengthen climate-adaptive regulation, optimize land use to enhance water retention, and plan development to reduce human pressure, ensuring sustainable utilization.

#### Main driving factors of carbon storage

**Fig. 10:**
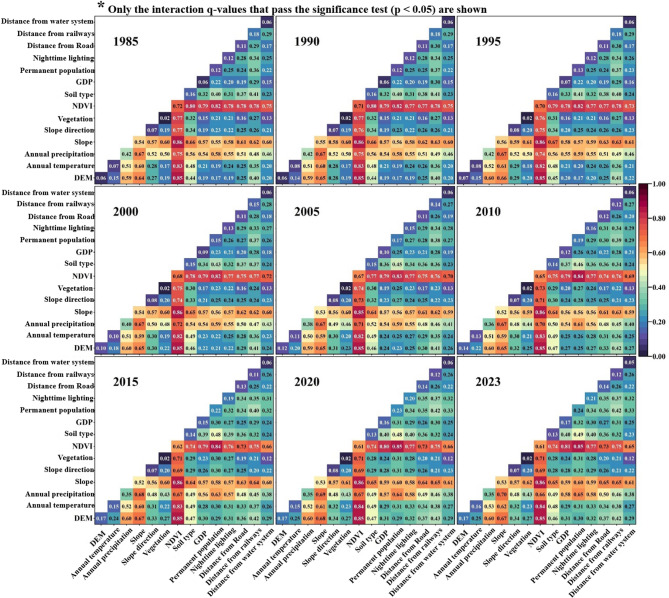
Interaction detection diagram of carbon storage driving factors.

According to the interaction detection results shown in Fig. 10, the driving factors of carbon storage in the middle reaches of the Yellow River from 1985 to 2023 exhibit significant spatiotemporal heterogeneity. Interactions among driving factors are primarily characterized by nonlinear enhancement and bivariate enhancement, with nonlinear enhancement accounting for 58.24–65.93% of cases. The results show that the interaction q-values tend to outweigh the isolated impact of single factors, demonstrating that alterations in carbon storage stem from the cooperative influence of multiple drivers. Overall, both natural and socioeconomic factors jointly influence the spatiotemporal patterns of carbon storage, with NDVI consistently showing strong interaction effects and emerging as a key regulator of carbon storage dynamics.

Between 1985 and 2000, carbon storage was mainly controlled by natural factors. Combinations such as slope ∩ NDVI (q = 0.86), DEM ∩ NDVI (q = 0.85), and mean annual temperature ∩ NDVI (q = 0.82–0.83) showed high explanatory power across multiple periods, indicating that topography and climate conditions played a critical role in the initial formation and variation of carbon storage. After 2005, the influence of socioeconomic factors gradually increased. Interactions such as NDVI ∩ population, NDVI ∩ GDP, and NDVI ∩ nighttime light index exhibited rising q-values, particularly NDVI ∩ population (q = 0.82–0.85) and NDVI ∩ GDP (q = 0.79–0.81), demonstrating that human activities increasingly impacted carbon storage alongside economic development and urban expansion. Additionally, the nighttime light index, representing human activity intensity, became a major component of driving factor combinations after 2005, reaching a high level in 2023 (q = 0.77), further highlighting the profound influence of urbanization on regional carbon storage. These findings indicate that carbon storage dynamics are jointly driven by topography, vegetation, climate, and human activities. Future management should strengthen vegetation restoration and optimize land use, control the impacts of urban expansion, and implement spatially differentiated protection measures to enhance the scientific basis of ecosystem management.

#### Trade-offs and synergies between water yield and carbon storage

##### Trade-offs and synergies between water yield and carbon storage

**Fig. 11:**
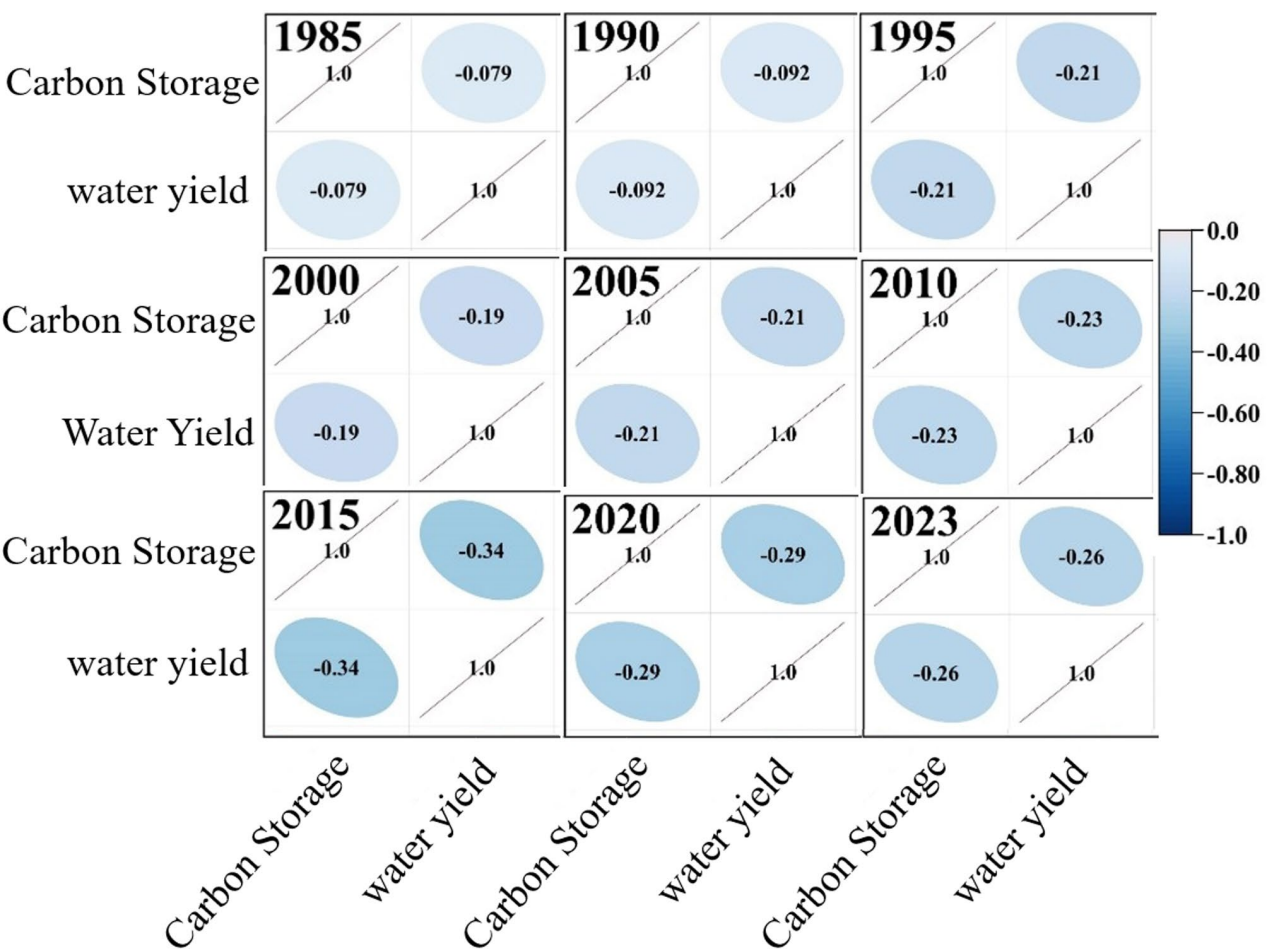
Correlation analysis of two ecosystem services in the study area from 1985 to 2023 (1985:*r* = − 0.079, p = n.s.; 1990: *r* = − 0.092,p = n.s.; 1995:*r* = − 0.21, *p* < 0.05; 2000: *r* = − 0.19, *p* < 0.01; 2005:*r* = − 0.21, *p* < 0.008; 2010: *r* = − 0.23, p = < 0.007; 2015: *r* = − 0.34, *p* = 0.005; 2020: *r*=− 0.29, *p* < 0.003;2 023: *r* = − 0.26,*p* < 0.001).

Figure 11 illustrates the temporal changes in the correlation between two key ecosystem services—water yield and carbon storage—in the middle reaches of the Yellow River from 1985 to 2023. Based on Pearson correlation analysis, the relationships between these ecosystem services exhibit significant spatiotemporal dynamics, revealing the trade-off and synergy mechanisms underlying ecosystem functionality.

To quantitatively define the trade-offs and synergies, we adopted the following classification based on the absolute value of the Pearson correlation coefficient (|r|): |r| < 0.1 indicates no significant correlation; 0.1 ≤ |r| < 0.2 indicates a weak correlation; 0.2 ≤ |r| < 0.3 indicates a moderate correlation; and |r| ≥ 0.3 indicates a strong correlation. A significant negative correlation (*p* < 0.05) is defined as a trade-off, while a significant positive correlation (*p* < 0.05) is defined as a synergy. This classification is widely used in ecological statistics to standardize the interpretation of correlation strength^46,47^.

Overall, water yield and carbon storage are predominantly negatively correlated, reflecting the competition between water supply and vegetation water demand. In particular, in the water-limited middle reaches of the Yellow River, vegetation restoration often increases evapotranspiration, thereby reducing water yield.

Temporally, after 2000, as human activities intensified and ecological protection policies were implemented, the trade-off relationship between ecosystem services became increasingly pronounced. The negative correlation between water yield and carbon storage strengthened significantly (*r* = − 0.29, *p* < 0.001), indicating that growing water demands driven by economic development exacerbated conflicts with ecological conservation.

##### Spatial heterogeneity of trade-offs and synergies

**Fig. 12:**
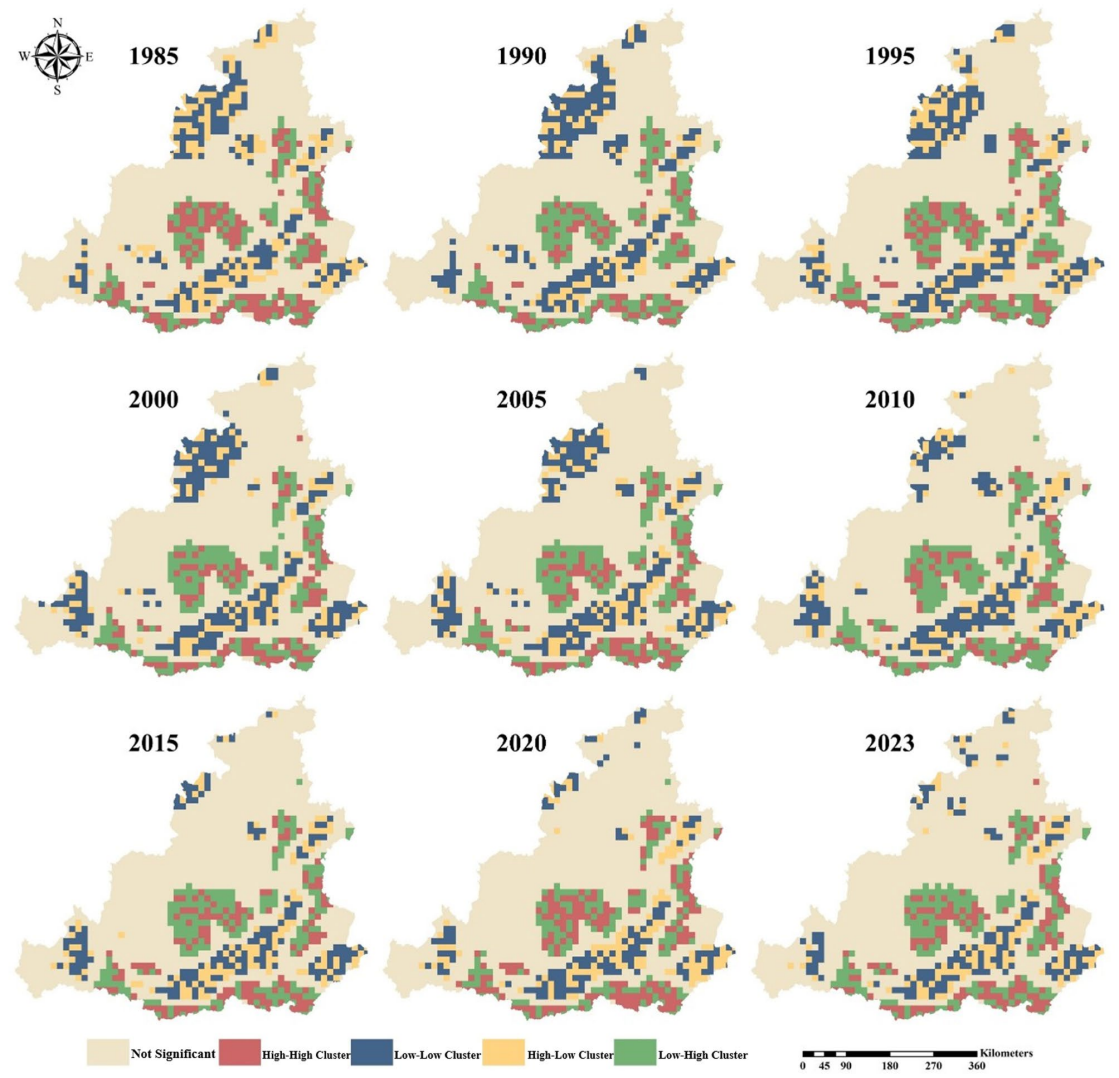
Water yield-carbon storage clustering diagram in the study area from 1985 to 2023 These maps were created using ArcGIS Pro 3.4 (https://www.esri.com/software/arcgis-pro). The spatial data sources are detailed in Table 1.

Analysis of the spatial clustering maps of water yield and carbon storage (Fig. 12) reveals the spatiotemporal distribution patterns and evolutionary trends of these two ecosystem services across the study area. From a spatial clustering perspective, high–high regions are primarily concentrated in areas with well-preserved ecosystems and high vegetation coverage. In these regions, both water yield and carbon storage are relatively high, indicating that ecological restoration and protection measures produce significant synergistic effects. Conversely, low–low regions are mainly distributed in areas with high land-use intensity, particularly those dominated by intensive agriculture and urbanization. In these areas, both water yield and carbon storage are low, reflecting the negative impacts of human activities on ecosystem services, particularly the dual depletion of water resources and carbon stocks. Additionally, high–low and low–high types are observed in Fig. 12, suggesting spatial mismatches between water yield and carbon storage across different times and locations. These mismatches are closely associated with land-use transitions, climate change, and the effectiveness of regional ecological protection policies.

**Fig. 13:**
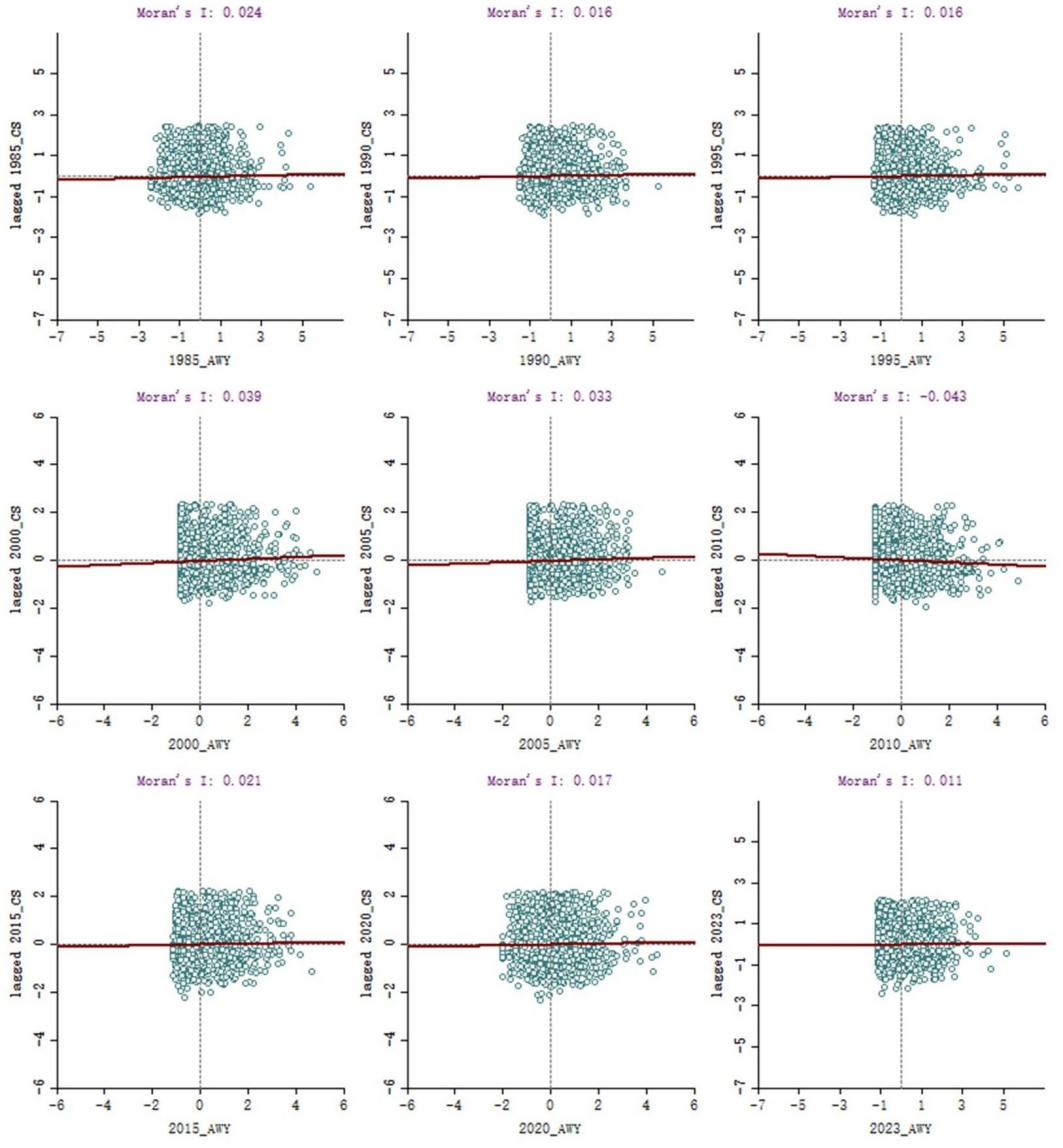
Water yield-carbon storage local Moran’s I scatter plot in the study area from 1985 to 2023.

Figure 13 further quantifies the spatial autocorrelation between water yield and carbon storage using bivariate local Moran’s I scatterplots. The Moran’s I index exhibits notable fluctuations over time. In 1985, Moran’s I was 0.024, indicating a weak positive correlation between water yield and carbon storage. In subsequent years, Moran’s I fluctuated and dropped to − 0.043 in 1995, reflecting an increase in negative correlation, particularly between high water-yield areas and low carbon storage areas, highlighting the spatial complexity and heterogeneity of ecosystem service functions within the basin. By 2023, although Moran’s I showed some recovery, overall spatial autocorrelation remained weak, suggesting that the spatial synergy between water yield and carbon storage had not yet been fully established.

By combining the local Moran’s I results in Fig. 13 with the spatial clustering map in Fig. 12, the geographical characteristics of different spatial association patterns can be more clearly identified. High–High (HH) clusters are mainly distributed in ecological conservation areas, such as the western Henan region and the Taihang Mountains, where forestland and grassland dominate and vegetation restoration projects are intensively implemented, resulting in consistently high levels of ecosystem services. In contrast, Low–Low (LL) clusters are primarily concentrated in highly urbanized areas, including parts of central Shaanxi and the Taiyuan urban agglomeration, characterized by extensive construction land and reduced ecological space.

High–Low (HL) and Low–High (LH) clusters are mainly located in transitional zones between urban expansion areas and ecological restoration regions, reflecting strong spatial heterogeneity and land-use conversion processes. These areas are often associated with mixed land-use patterns, such as cropland–construction land mosaics, indicating potential instability in ecosystem service provision.

## Discussion

### Comprehensive driving mechanisms of land use change

Although economic development and policy interventions were identified as major drivers of land use change in the study area, additional factors also play important roles and should be acknowledged to provide a more comprehensive understanding of the driving mechanisms. It should be noted that the impacts of ecological policies are not uniform over time. The implementation intensity of the Grain-for-Green Program was significantly higher during its initial expansion stage (2000–2006) than in the post-2010 consolidation period, resulting in distinct differences in land-use transition rates and ecosystem service responses across stages.

First, climate change has significantly influenced land cover transitions, particularly in semi-arid regions where increasing temperature and fluctuating precipitation affect vegetation productivity, soil moisture, and the stability of cultivated and grassland ecosystems. Second, population migration and the redistribution of labor resources—including urbanization, rural depopulation, and the shift from agricultural to non-agricultural employment—have reshaped land demand, leading to both farmland abandonment in marginal areas and urban expansion in economic centers. Third, technological progress, such as improvements in agricultural water-saving technologies, land consolidation, remote sensing monitoring, and ecological restoration techniques, has altered land use efficiency and enhanced the effectiveness of ecological projects.

These factors interact with socio-economic development and policy incentives, jointly shaping the spatial heterogeneity and temporal dynamics of land use change.

### Exploring the impact mechanism of land use change on water yield and carbon storage

In the context of fast urban expansion, changes in land use represent an important determinant of ecosystem organization and functioning. The integrated InVEST-PLUS-GeoDetector framework developed in this study offers a mechanistic pathway from land-use change simulation to ecosystem service impact and, crucially, to the diagnosis of underlying drivers. The integrated InVEST–PLUS–GeoDetector framework enabled the diagnosis of how land-use transitions affect water yield and carbon storage and allowed the identification of interacting driving factors.

Synergies and trade-offs among ecosystem services in the study area are fundamentally governed by vegetation-mediated ecological and hydrological processes. Increased vegetation coverage enhances carbon storage and soil retention through higher photosynthetic carbon sequestration, strengthened root–soil interactions, and improved soil organic matter accumulation, thereby promoting synergies among regulating services. However, the same increase in vegetation biomass intensifies evapotranspiration and canopy interception, which reduces effective water yield despite improvements in carbon storage and soil stability^48^. As a result, regions with dense vegetation often exhibit strong synergies in carbon-related and soil-related services, accompanied by pronounced trade-offs in water provision.

Land use transitions associated with urban expansion constitute another major driver of trade-offs. The conversion of natural or cultivated land to impervious surfaces interrupts soil carbon input pathways, eliminates vegetation-related carbon sequestration, and accelerates surface runoff, leading to reductions in both carbon storage and soil retention.

Overall, synergies arise primarily from vegetation-mediated ecological processes, whereas trade-offs emerge from hydrological constraints and land conversion impacts. Understanding these intrinsic mechanisms provides a process-based basis for interpreting the spatial patterns of ecosystem service interactions observed in the study.

### Exploring the trade-offs and synergies between water yield and carbon storage

This study further investigates the synergies and trade-offs between water yield and carbon storage in the Middle Yellow River Basin, and examines how land use dynamics influence their interactions. The results show that in ecologically favorable areas such as western Henan and the Taihang Mountains, higher vegetation coverage enhances both water regulation and carbon sequestration, leading to strong positive correlations between the two services. This finding is consistent with domestic and international research, which highlights that forest conservation and restoration projects often simultaneously improve water yield and carbon storage.

However, accelerated urbanization has driven substantial land-use transformations in parts of the basin, particularly through the expansion of construction land. The growth of built-up areas reduces available habitats and alters land-cover composition, thereby expanding low-carbon storage zones and weakening the stability of carbon sequestration. Such transformations also intensify negative trade-offs among ecosystem services by constraining ecological functions. For example, in central Shanxi, the continuous reduction of cropland and rapid urban expansion, while supporting short-term economic growth, have resulted in long-term declines in both water yield and carbon storage, highlighting the ecological costs associated with urban sprawl.

Land-use change profoundly reshapes the interaction between water yield and carbon storage, with urbanization weakening synergies and amplifying trade-offs, a pattern also observed in other rapidly developing regions such as the Yangtze River Basin and the Amazon. Meanwhile, marked spatial heterogeneity exists within the basin: ecological restoration in western Henan and the Taihang Mountains enhances synergies, whereas highly urbanized areas of Shaanxi and Taiyuan experience intensified trade-offs due to cropland loss and construction land expansion. These contrasts underscore the need for region-specific land-use planning and ecological management strategies.

### Recommendations for enhancing ecosystem services in the study area

Based on the spatial heterogeneity of land use change and ecosystem service patterns, three management zones were identified to guide targeted regional governance: ecological conservation priority zones, ecological restoration and control zones, and coordinated development and utilization zones. Ecological conservation priority zones are mainly distributed in areas with high elevation and steep slopes, where ecological service values are high. In these zones, management should strictly limit construction activities and land conversion, prioritize afforestation, grassland rehabilitation, and soil–water conservation projects, and strengthen ecological redline protection alongside long-term monitoring. Ecological restoration and control zones, in contrast, include regions characterized by severe land degradation and low vegetation cover. For these areas, ecological restoration projects such as slope stabilization, re-vegetation, and gully treatment are recommended. Additionally, agricultural expansion should be controlled, intensive land use reduced, and water-saving agriculture along with sustainable land management practices should be promoted.

Coordinated development and utilization zones are located in flat areas with moderate ecological service values and good accessibility. In these zones, compact urban development should be encouraged to avoid excessive land expansion, and industrial layout should be guided toward low-carbon and green industries. It is also essential to balance land development with ecological protection by incorporating ecological service values into spatial planning. These zoning-based management strategies provide a practical framework for regional planning and highlight the need to balance ecological protection, restoration, and sustainable development according to the spatial heterogeneity revealed in this study.

### Prospects

This study provides valuable theoretical support and technical guidance for watershed ecological protection and sustainable development. Despite meaningful progress, there remains considerable scope for future research, particularly in the following areas:

1. Improving data accuracy for ecosystem service assessments: The present analysis relied on existing remote sensing data and land-use classifications, which, while informative, are subject to limitations in resolution and classification accuracy. Future studies should incorporate higher-resolution remote sensing imagery and more refined classification methods to enhance spatial and temporal precision, thereby providing more reliable datasets for detailed ecosystem service evaluations.
2. Model Limitations Related to Parameterization: As the InVEST model was originally developed using global datasets, its default biophysical parameters may not fully capture the hydrological processes of the arid–semi-arid transition zone in the Middle Yellow River Basin. Despite the localized parameter adjustments implemented in this study, uncertainties remain due to spatial heterogeneity of soil water-holding capacity, vegetation water consumption differences, and the highly variable precipitation regime. These factors may lead to underestimation or overestimation of water yield in some sub-regions. Future work should incorporate field-measured soil moisture data, root distribution measurements, and calibration against observed runoff to further reduce parameter uncertainty.
3. Model Limitations in Arid and Semi-arid Regions: Although the modeling framework provides an effective tool for assessing land use change and ecosystem services, its applicability in arid and semi-arid regions is subject to several limitations. First, the simplified hydrological assumptions of the InVEST water-related modules may not fully capture the strong seasonal variability, low soil moisture content, and rapid infiltration processes typical of dryland environments. These conditions often lead to discrepancies between simulated and actual water yield or soil conservation values. Second, the sparse vegetation cover and high heterogeneity of soil properties in semi-arid landscapes reduce the accuracy of parameterization, especially when default or generalized biophysical parameters are used. Third, the PLUS model, while effective in simulating land expansion patterns, may not fully reflect the complex interactions between climate constraints, water scarcity, and socio-economic drivers that shape land use dynamics in dry regions.

Therefore, the results should be interpreted with caution, and future studies may incorporate field measurements, finer-resolution soil moisture data, or region-specific parameters to improve model reliability in arid and semi-arid contexts.

## Conclusions

This study integrates the land-use transfer matrix, InVEST model, PLUS model, GeoDetector model, and correlation analysis to systematically characterize the spatial patterns and driving mechanisms of land-use change, water yield, and carbon storage in the middle reaches of the Yellow River Basin. Furthermore, it explores the impacts of land-use transitions on water yield and carbon storage dynamics. The major conclusions are as follows:

1. Characteristics and driving mechanisms of land-use transitions: Between 1985 and 2023, land use in the middle Yellow River Basin underwent significant changes, characterized by a decline in cropland and an expansion of forestland and construction land. Ecological restoration projects led to concentrated forest and grassland recovery in designated restoration zones. These transitions were jointly driven by climate change, population growth, economic development, and ecological policies, exhibiting marked spatial heterogeneity. They reflect the dual effects of ecological restoration and urbanization. Moving forward, priority should be given to the protection and restoration of ecologically fragile areas, alongside scientific planning of urbanization and cropland preservation, to foster coordinated economic and ecological development.
2. Spatiotemporal dynamics of water yield and carbon storage: Water yield showed significant fluctuations, influenced by the trade-offs between water resource regulation and vegetation restoration. Carbon storage varied markedly across land-use types, with forests exhibiting the highest carbon storage, followed by cropland and grassland, while construction land, unused land, and water bodies maintained relatively low levels due to limited vegetation cover and carbon sequestration capacity. Overall, ecological conservation and restoration measures have positively contributed to ecosystem functionality. However, the protection and rehabilitation of low-quality areas remain insufficient. In particular, challenges persist regarding rational water resource use and soil conservation, necessitating more targeted management measures.
3. Synergies and trade-offs among ecosystem services: Water yield and carbon storage exhibit both synergies and trade-offs. With accelerating urbanization, human activities increasingly intensify the negative impacts on ecosystem services, underscoring the critical role of ecological conservation measures in mitigating these effects.

## Data Availability

The datasets used and/or analysed during the current study available from the corresponding author on reasonable request.

## Appendix

See Tables 6, 7, 8, 9, 10, 11, 12 and 13.

**Table 6:** Land use transfer matrix from 1985 to 1990 (hm²).

1985	1990						
	Cropland	Forest	Crassland	Water	Impervious	Barren	Transfer Out
Cropland	1,316,227	73,839.51	498,059.64	8170.74	66,248.46	13.59	646,331
Forest	73,231.65	6,430,609	46,789.02	151.20	656.73	0.00	120,828.60
Crassland	385,065.9	60,602.22	12,401,966	890.73	1282.50	50,119	497,960.9
Water	5318.91	43.56	1876.05	107,052	1134.72	42.93	8416.17
Impervious	25,778.34	346.59	1078.29	3020.94	417,233	10.08	30,234.24
Barren	26.37	0.00	14,938.47	55.98	26.19	533,455	15,047.01
Transfer In	489,421.2	134,831.8	562,741.47	12,289.59	69,348.6	50,186	1,318,818.8

**Table 7:** Land use transfer matrix from 1990 to 1995 (hm²).

1990	1995						
	Cropland	Forest	Crassland	Water	Impervious	Barren	Transfer Out
Cropland	12,194,444	39,481.74	1,282,242	7380.18	128,069.8	72.09	1,457,246
Forest	79,756.29	6,445,501.0	40,075.74	0.09	108.45	0.00	119,940.5
Crassland	1,178,906.9	190,008.9	11,475,100	3969.1	8599.86	108,122	1,489,607
Water	17,574.12	76.14	2468.07	89,663	9452.97	107.19	29,678.49
Impervious	107.10	0.09	11.07	2521.71	483,941.79	0.81	2640.78
Barren	894.69	0.00	83,554.47	497.25	934.56	497,760.	85,880.9
Transfer In	1,277,239	229,566.8	1,408,352	14,368.4	147,165.6	108,302.9	3,184,995

**Table 8:** Land use transfer matrix from 1995 to 2000 (hm²).

1995	2000						
	Cropland	Forest	Crassland	Water	Impervious	Barren	Transfer Out
Cropland	11,657,034	127,348.5	1,497,636.9	12,833.1	176,784.4	46.26	1,814,649.4
Forest	107,765.9	6,390,553	175,077.6	389.16	1281.69	0.18	284,514.57
Crassland	1,056,244	329,358.6	11,455,744	2796.6	21,120.84	18,188.01	1,427,708.1
Water	17,307.99	224.10	2784.87	72,459.	10,885.32	369.45	31,571.73
Impervious	68,730.03	999.36	13,707.45	6676.65	540,932.3	61.65	90,175.14
Barren	1501.56	0.00	191,896.5	334.98	1570.50	410,759	195,303.6
Transfer In	1,251,549	457,930	1,881,103	23,030	211,642.8	18,665.55	3,843,922

**Table 9:** Land use transfer matrix from 2000 to 2005 (hm²).

2000	2005						
	Cropland	Forest	Crassland	Water	Impervious	Barren	Transfer Out
Cropland	1,119,643	136,237	1,358,651	20,999	196,214.5	49.14	1,712,152
Forest	92,777.1	6,564,666	189,240.75	556.38	1242.54	0.90	283,817.70
Crassland	1,118,870	317,897	11,843,174	4078.8	27,178.11	25,648.47	1,493,673.4
Water	9382.95	419.67	1879.02	77,505	6234.93	68.76	17,985.33
Impervious	80,943.6	1057.14	12,690.18	12,143	645,693.1	47.34	106,882.02
Barren	712.71	0.18	138,302.8	647.01	1733.85	288,028	141,396.5
Transfer In	1,302,686	455,612.04	1,700,763	38,425	232,604.01	25,814.6	3,755,907

**Table 10:** Land use transfer matrix from 2005 to 2010 (hm²).

2005	2010						
	Cropland	Forest	Crassland	Water	Impervious	Barren	Transfer Out
Cropland	10,490,926	200,370	1,505,326.8	18,887.3	283,491.6	116.28	2,008,192
Forest	118,324.98	6,532,346	365,894.10	1141.38	2570.13	1.26	487,931.8
Crassland	1,201,072.9	525,451.3	11,743,608	4226.49	50,372.01	19,206.81	1,800,329
Water	13,810.68	531.45	2435.04	88,528.4	10,351.89	273.69	27,402.75
Impervious	142,075.26	1125.45	29,583.90	11,868	693,507.2	137.25	184,789.8
Barren	586.26	0.90	154,966.32	302.67	1431.18	156,556	157,287.3
Transfer In	1,475,870	727,479	2,058,206	36,425	348,216.84	19,735.2	4,665,933

**Table 11:** Land use transfer matrix from 2010 to 2015 (hm²).

2010	2015						
	Cropland	Forest	Crassland	Water	Impervious	Barren	Transfer Out
Cropland	9,901,896.7	176,760.3	1,552,215.4	18,207.2	317,522.6	194.13	2,064,899.7
Forest	169,160.13	6,725,497	361,585.89	787.32	2773.53	20.88	534,327.75
Crassland	1,257,082.2	648,321.3	11,824,951	4972.59	52,019.46	14,467.9	1,976,863.5
Water	14,148.99	1283.04	2574.72	94,259.2	12,485.07	203.22	30,695.04
Impervious	169,088.67	2284.74	31,247.55	11,216.3	827,640.9	245.79	214,083.09
Barren	1962.63	0.90	95,540.85	177.93	959.04	77,650.1	98,641.35
Transfer In	1,611,442	828,650	2,043,164	35,361	385,759.71	15,131	4,919,510.6

**Table 12:** Land use transfer matrix from 2015 to 2020 (hm²).

2015	2020						
	Cropland	Forest	Crassland	Water	Impervious	Barren	Transfer Out
Cropland	9,808,989	216,902.	1,199,737.	13,740.84	273,573.9	395.10	1,704,350
Forest	207,340.5	7,038,151	304,356.4	1237.05	3026.97	35.82	515,996.82
Crassland	1,352,085.6	665,464.7	11,766,967	3331.35	53,057.43	27,208.8	2,101,148.1
Water	12,532.59	1096.92	2483.10	104,309	8921.34	277.47	25,311.42
Impervious	181,558.5	2575.89	26,000.19	13,348.8	989,575.8	341.37	223,824.8
Barren	1528.74	13.05	31,526.37	235.98	1093.77	58,384.	34,397.91
Transfer In	1,755,046	886,053	1,564,103	31,894.1	339,673.50	28,258	4,605,029

**Table 13:** Land use transfer matrix from 2020 to 2023 (hm²).

2020	2023						
	Cropland	Forest	Crassland	Water	Impervious	Barren	Transfer Out
Cropland	9,946,535	276,363	1,062,728.1	11,942.1	266,149.8	316.35	1,617,499
Forest	271,932	731,968	325,740.06	1832.85	4995.00	24.03	604,524.1
Crassland	1,305,985	539,759	11,418,095	2720.07	45,279.99	19,230.9	1,912,975.9
Water	16,579.2	1144.44	2558.25	105,729	10,066.14	125.91	30,474.00
Impervious	225,393	2761.47	28,858.50	9405.9	1,062,308	521.19	266,940.54
Barren	1004.76	33.12	32,814.45	121.05	2278.35	50,391.09	36,251.73
Transfer In	1,820,894	820,061	1,452,699	26,021.9	328,769.28	20,218.41	44,68,665.87
